# Functional significance of rare neuroligin 1 variants found in autism

**DOI:** 10.1371/journal.pgen.1006940

**Published:** 2017-08-25

**Authors:** Moe Nakanishi, Jun Nomura, Xiao Ji, Kota Tamada, Takashi Arai, Eiki Takahashi, Maja Bućan, Toru Takumi

**Affiliations:** 1 RIKEN Brain Science Institute, Wako, Saitama, Japan; 2 Graduate School of Biomedical Sciences, Hiroshima University, Minami, Hiroshima, Japan; 3 Department of Genetics, Perelman School of Medicine, University of Pennsylvania School of Medicine, Philadelphia, Pennsylvania, United States of America; 4 Genomics and Computational Biology Graduate Group, Perelman School of Medicine, University of Pennsylvania, Philadelphia, Pennsylvania, United States of America; 5 Department of Psychiatry, Perelman School of Medicine, University of Pennsylvania School of Medicine, Philadelphia, Pennsylvania, United States of America; Pennsylvania State University, UNITED STATES

## Abstract

Genetic mutations contribute to the etiology of autism spectrum disorder (ASD), a common, heterogeneous neurodevelopmental disorder characterized by impairments in social interaction, communication, and repetitive and restricted patterns of behavior. Since *neuroligin3* (*NLGN3*), a cell adhesion molecule at the neuronal synapse, was first identified as a risk gene for ASD, several additional variants in *NLGN3* and *NLGN4* were found in ASD patients. Moreover, synaptopathies are now known to cause several neuropsychiatric disorders including ASD. In humans, NLGNs consist of five family members, and *neuroligin1* (*NLGN1*) is a major component forming a complex on excitatory glutamatergic synapses. However, the significance of *NLGN1* in neuropsychiatric disorders remains unknown. Here, we systematically examine five missense variants of *NLGN1* that were detected in ASD patients, and show molecular and cellular alterations caused by these variants. We show that a novel *NLGN1* Pro89Leu (P89L) missense variant found in two ASD siblings leads to changes in cellular localization, protein degradation, and to the impairment of spine formation. Furthermore, we generated the knock-in P89L mice, and we show that the P89L heterozygote mice display abnormal social behavior, a core feature of ASD. These results, for the first time, implicate rare variants in *NLGN1* as functionally significant and support that the NLGN synaptic pathway is of importance in the etiology of neuropsychiatric disorders.

## Author summary

Autism spectrum disorder (ASD) is a childhood disorder manifested by abnormal social behavior, interests, and activities. The genetic contribution to ASD is higher than in other psychiatric disorders such as schizophrenia and mood disorders. Here, we found a novel mutation in *NLGN1*, a gene encoding a synaptic protein, in patients with ASD. We also developed a mouse model with this mutation, and showed that the model mouse exhibits abnormal social behavior. These results suggest that a rare variant in *NLGN1* is functionally significant and support that the NLGN synaptic pathway may be important in the etiology of neuropsychiatric disorders. This humanized mouse model recapitulates some of the symptoms of patients with ASD and will serve as a valuable tool for therapeutic development.

## Introduction

Autism spectrum disorder (ASD) is a common, heterogeneous neurodevelopmental disorder characterized by impairments in social interaction, communication, and repetitive and restricted patterns of behavior. The Centers for Disease Control and Prevention (CDC) has recently reported that the prevalence of ASD is 1:68 [1]. ASD is highly-heritable, but the genetic basis is complex, and numerous copy number variants (CNVs) and single nucleotide variants (SNVs) have been identified in patients with ASD [2–8]. Recent large-scale genetic studies have highlighted hundreds of genes as risk factors for ASD pathogenesis [2, 3, 7]. Many of these risk-genes have a role in synaptic function and development [2, 9].

Neuroligins (NLGNs) are well-characterized as postsynaptic cell-adhesion molecules, composed of five family members in humans (*NLGN1*, *2*, *3*, *4X* and *4Y*) [10, 11]. Among NLGN family molecules, *NLGN3* and *NLGN4* were initially associated with non-syndromic ASD, with less support for the relevance of *NLGN1* and *NLGN2* [12–14]. NLGN family members share the domain structure that comprises a large N-terminal extracellular region containing an esterase homology domain and a C-terminal cytoplasmic tail, separated by a single transmembrane region [15]. NLGN proteins form a trans-synaptic complex with presynaptic Neurexin (NRXN) via the extracellular domain, whereas the cytoplasmic domain interacts with postsynaptic molecules including PSD95, SHANK, EPAC, and MDGA [10, 11, 16, 17]. Genetic variants in *NRXN* and *SHANK* family members have been recurrently identified in patients with ASD as well as other neuropsychiatric disorders [18–21]. Moreover, CNVs and SNVs in binding partners of *NLGN1*, *EPAC2*, and *MDGA2* have been reported in ASD [22, 23]. Thus, although the specific synaptic pathway for etiology of ASD is not fully understood, the NRXN-NLGN complex and its downstream cascades have been suggested in processes underlying cognition and social behavior.

Several variants in *NLGN3* and *NLGN4* identified in patients with ASD have been well characterized. Variants in these genes result in loss- or gain-of-functions. The reported missense variant in *NLGN4* (R87W) leads to a loss-of-function, whereas a variant in *NLGN3* (R451C) acts in a gain-of-function manner [24, 25]. A variant in *NLGN4* (R704C) is also reported to modulate synaptic response through altering surface AMPA receptor levels [26–28]. Several types of dysfunction in NLGNs might be involved in ASD pathogenesis. Of note, mouse models with these variants show abnormal behavior relevant to ASD, further supporting the role of these genes in the etiology of ASD [25, 28]. Altered expression of *Nlgn1* in the brain has been observed in several mouse models for ASD, such as *Eif4ebp2* and *Fmr1* knockout mice [29, 30]. Moreover, normalizing the expression of *Nlgn1* in these mutants ameliorates their ASD-like behavior, indicating that changes in NLGN1 dosage may lead to behavioral abnormalities. However, mouse mutants with *Nlgn1* variants observed in ASD subjects have not been reported, and none of the pathogenic variants have ever been functionally evaluated. Thus the significance of *NLGN1* in neuropsychiatric disorders is still elusive.

In the present study, we systematically examined five rare missense variants in *NLGN1*, including a novel variant found in patients with ASD, to elucidate the molecular and cellular alteration caused by these variants. Furthermore, we developed a model mouse that reflects a novel *NLGN1* variant, Pro89Leu (P89L), identified by exome sequencing of two patients with ASD, and we analyzed behavioral outcomes in this knock-in (KI) mouse line.

## Results

### Identification of P89L substitution in *NLGN1*

**Fig 1:**
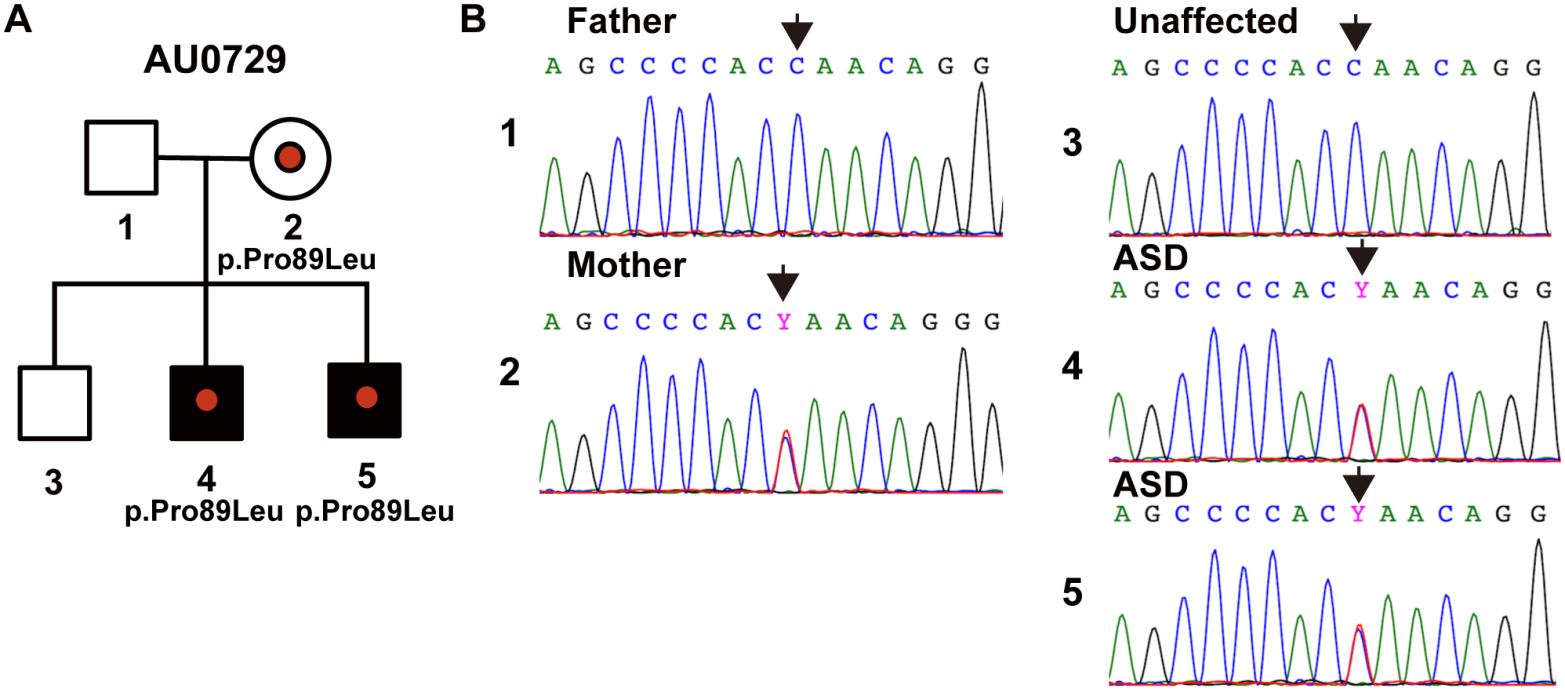
Identification of P89L substitution in *NLGN1*. (A) Pedigree of the family harboring the *NLGN1* c.266C>T (p.Pro89Leu) substitution. (B) Sequence electropherograms of family AU0729. Father (1), Mother (2), unaffected sibling (3), affected siblings (4 and 5). Arrows indicate the location of the *NLGN1* c.266C>T substitution.

*NLGN1* is highly expressed in the brain through prenatal and postnatal stages (S1A Fig), and based on the analysis of genetic variation in exomes of ~60,000 subjects in the Exome Aggregation Consortium (ExAC), this gene is intolerant to deleterious and loss-of-function variants as determined by the residual variation intolerance score (RVIS) and the probability of being loss-of-function intolerant (pLI) (RVIS percentile = 7.3, pLI percentile = 23.0) [31, 32]. We performed exome sequencing of a family (AU0729) from the Autism Genetic Resource Exchange (AGRE) [33] with a pair of siblings affected with a non-syndromic autism (AU072904, AU072905) (Fig 1A). To prioritize variants for experimental validation, we defined these criteria as following: a) nonsynonymous or stop-gain; b) absent in ExAC data set (version 3), NHLBI Exome Sequencing Project (ESP6500 version 2, European ancestry) and 1000 Genomes [34] (2014 Oct, European ancestry); c) Combined Annotation Dependent Depletion (CADD) [35] phred-scale score>10 and d) gene-level Residual Variation Intolerance Score (RVIS) [31] < 50^th^ percentile. Exome sequencing of the AGRE family (AU0729) identified variants in seven genes (*NLGN1*, *RFX1*, *PPP2R5C*, *C2CD5*, *NCAM2*, *ZBTB1*, and *DISP1*) that were shared by both affected siblings and fulfilled the above listed criteria (S1 Table). Among the seven variants, two variants in *NLGN1* and *DISP1* were absent in the unaffected sibling (Fig 1B and S1 Table). As *NLGN1* is a synaptic molecule, with paralogs (*NLGN3* and *NLGN4*) implicated in ASD, and based on *in silico* evaluation of other variants (S1 Table), we selected the *NLGN1* variant (P89L) for further *in silico* analysis and experimental validation, although we cannot exclude the possibility that other heterozygous deleterious variants may lead to ASD susceptibility. Sanger sequencing confirmed that the *NLGN1* variant (P89L) was inherited from the mother, who was not diagnosed with ASD but suffered from obsessive-compulsive disorder (OCD) and anxiety disorder (Fig 1B).

To further assess the functional significance of *NLGN1* variants to ASD etiology, we identified three rare missense variants in *NLGN1* (L269P, G297E, and H795Y) among the exonic variants of ~2,500 ASD families in the Simons Simplex Collection (SSC) [3, 36] and one missense variant in *NLGN1* (T90I) in two out of 362 ASD children recruited at Children's Hospital of Philadelphia (CHOP) (Table 1). Among the five *NLGN1* variants, three variants (P89L, L269P, and G297E) were novel and not detected in ExAC data set, and two variants (H795Y and T90I) were found in ExAC data set at an extremely low minor allele frequency (MAF = 0.000008 for H795Y, MAF = 0.00008 for T90I). All *NLGN1* variants identified in ASD subjects were heterozygous.

**Table 1:** Summary of extremely rare NLGN1 variants found in ASD.

No.	Proband ID (Collection)	sex	Position (hg19)	Alt (Ref)	Amino acid Change	Inheritance
1	AU072904 (AGRE)	M	Chr3: 173322654	T (C)	p.Pro89Leu	Maternal
2	AU072905 (AGRE)	M				
3	13876.p1 (SSC)	M	Chr3: 173993264	C (T)	p.Leu269Pro	Paternal
4	11045.p1 (SSC)	M	Chr3: 173996681	A (G)	p.Gly297Glu	Maternal
5	12157.p1 (SSC)	M	Chr3: 173999004	T (C)	p.His795Tyr	*de novo*
6	NA (CHOP)	NA	Chr3: 173322657	T (C)	p.Thr90Ile	NA
7	NA (CHOP)	NA				NA

NA, not available.

### *In silico* evaluation of *NLGN1* variants

To evaluate the pathogenic effect of the five detected *NLGN1* variants in ASD patients, we first performed *in silico* prediction. We applied seven tools (CADD [35], SIFT [37], PolyPhen2 [38], MutationTaster [39], LRT [40], Mutation Assessor [41], and FATHMM [42]) for the consensus prediction of deleterious effect. Almost all tools predicted three of the five variants (P89L, L269P, and G297E) as deleterious with highest probability (Table 2). However, only a subset of tools (SIFT, Mutation Taster, and LRT) predicted H795Y and T90I (Mutation Taster and LRT) as deleterious. Based on these results, we grouped these mutants into two types of pathogenic variants: high-risk variants (P89L, L269P, and G297E) and low-risk variants (H795Y and T90I).

**Table 2:** *In silico* evaluation of NLGN1 variants.

Amino acid change	MAF			*in silico* prediction								
	1000 Genomes	ESP 6500	ExAc	CADD	SIFT	Poly-Phen2	Mutation Taster	LRT	Mutation Assessor	FATHMM	Number of "deleterious"	Classification
P89L	NA	NA	NA	22.2	D	D	D	D	H	D	**7**	**High-risk**
L269P	NA	NA	NA	20.9	D	D	D	D	H	T	**6**	**High-risk**
G297E	NA	NA	NA	22.8	D	D	D	D	H	D	**7**	**High-risk**
H795Y	NA	NA	0.000008	13.6	D	B	D	D	M	T	**3**	**Low-risk**
T90I	NA	NA	0.00008	5.7	T	B	D	D	N	T	**2**	**Low-risk**

MAF: minor allele frequency. 1000 genome European ancestry and ESP 6500 European ancestry are used.

D and H indicate the highest probability (e.g. damaging or deleterious),

B, N and T indicate the lowest probability (e.g. benign or neutral or tolerated),

M indicates the intermediate probability of pathogenic effect, respectively.

We defined the variant as “deletrious” if 1) the variant was not found in 1000 Genomes, ESP 6500 and ExAc, 2) CADD score > 20, 3) the evaluation by each *in silico* tool was “D” or “H”.

**Fig 2:**
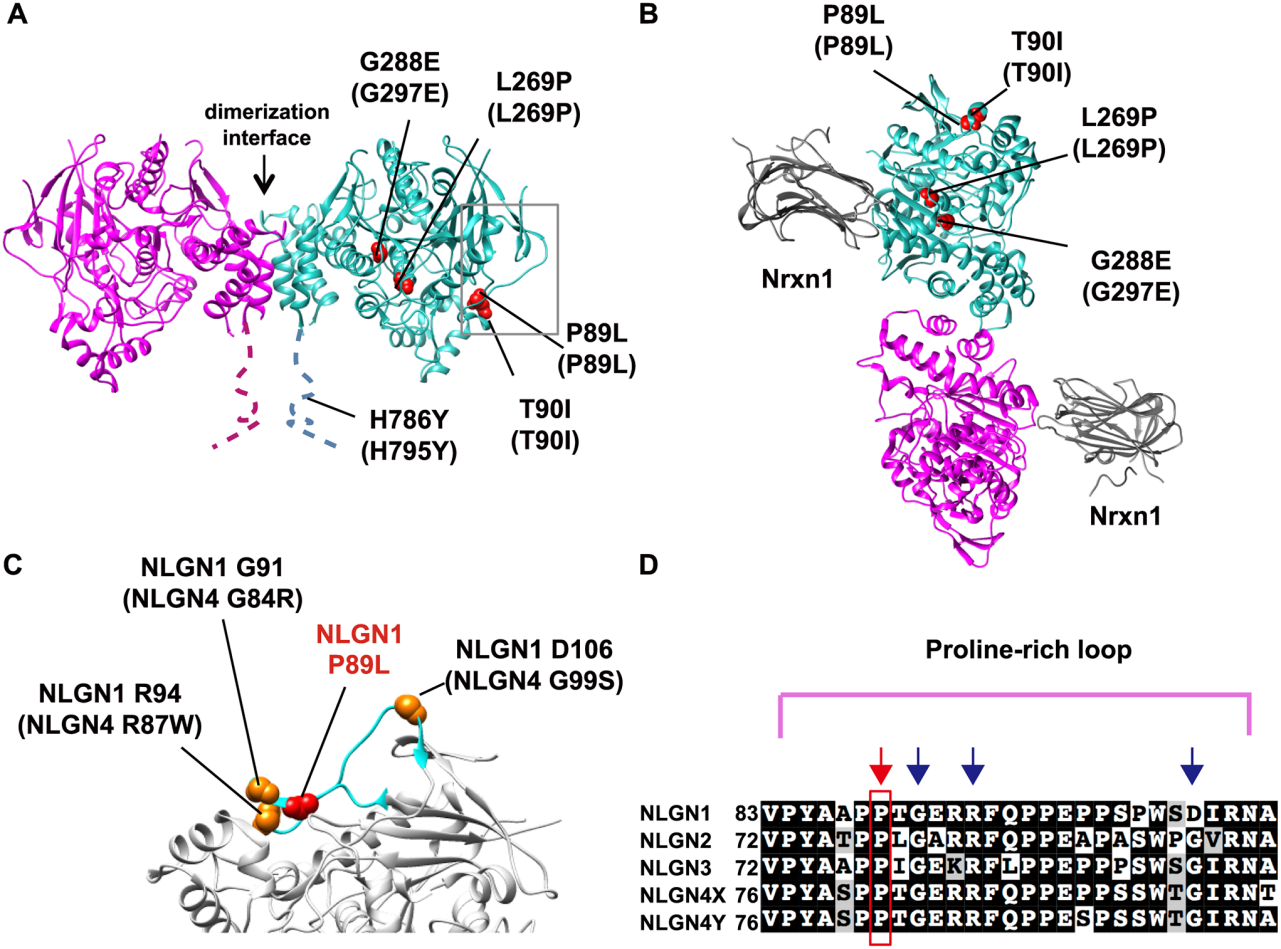
*In silico* investigation of *NLGN1* variants observed in patients with ASD. (A) Ribbon diagram of the extracellular part of the mouse NLGN1 dimer viewed from the side, based on Protein Data Bank (PDB) entry 3B3Q. The dotted line shows the NLGN1 region for which crystal structure is unavailable. Amino-acid numberings of mouse NLGN1 (human NLGN1) are indicated. The variants assessed in this study are shown in red. The gray box indicates the location of a proline-rich loop of NLGN1. (B) Ribbon diagram of the NLGN1-NRXN1β complex, viewed from the post-synaptic membrane with variants. (C) Enlarged image of the proline-rich loop structure of NLGN1 in which P89 is located (shown in light blue). Previously identified *NLGN4* missense variants (G84R, R87W, and G99S) found in ASD are also indicated. (D) The protein alignment of the NLGN1-4 family. The highly conserved P89 residue, mutated in ASD patients, is boxed in red, and pathogenic *NLGN4* variants, localized in the same loop, are shown as blue arrows.

In addition, we investigated the impact of these variants on the *NLGN1* structure and function, and mapped them on the crystal structure of mouse NLGN1 [43]. Four *NLGN1* variants (P89L, T90I, L269P, and G297E) were located in the extracellular esterase-homology domain (α/β-hydrolase fold domain) and one variant (H795Y) was located in the cytoplasmic tail (Fig 2A). None of the variants was localized in the known binding interface of *NLGN1*, thus these predicted deleterious variants likely have effects on function that is independent of binding with *NLGN1*-partner molecules (e.g. Neurexins) (Fig 2B). The amino-acid substitution in the esterase-homology domain can perturb the structure of the protein [44]. Particularly, P89L and T90I affected the proline-rich loop of the esterase-homology domain, which is structurally rigid and where three pathogenic variants of *NLGN4* observed in ASD subjects (G84R, R87W, and G99S) are located (Fig 2C). Its disruption by amino-acid substitution was suggested to cause protein misfolding [13, 24]. The *NLGN1* (P89L) site was perfectly conserved in vertebrates and NLGN family members (paralogs), further supporting the damaging effect on protein structure (Fig 2D). However, T90I was poorly conserved among NLGN paralogs, and therefore an amino-acid substitution may have a less severe effect. Another *NLGN1*-variant (L269P) located in a helix structure also likely affects the helix formation and structure of the NLGN1 protein. The destabilizing effect for the NLGN1 structure of variants found in ASD (P89L, L269P, and G297E) was further supported by FoldX calculation of free energy change [45] (S1B Fig).

### Expression analysis of *NLGN1* variants observed in patients with ASD

**Fig 3:**
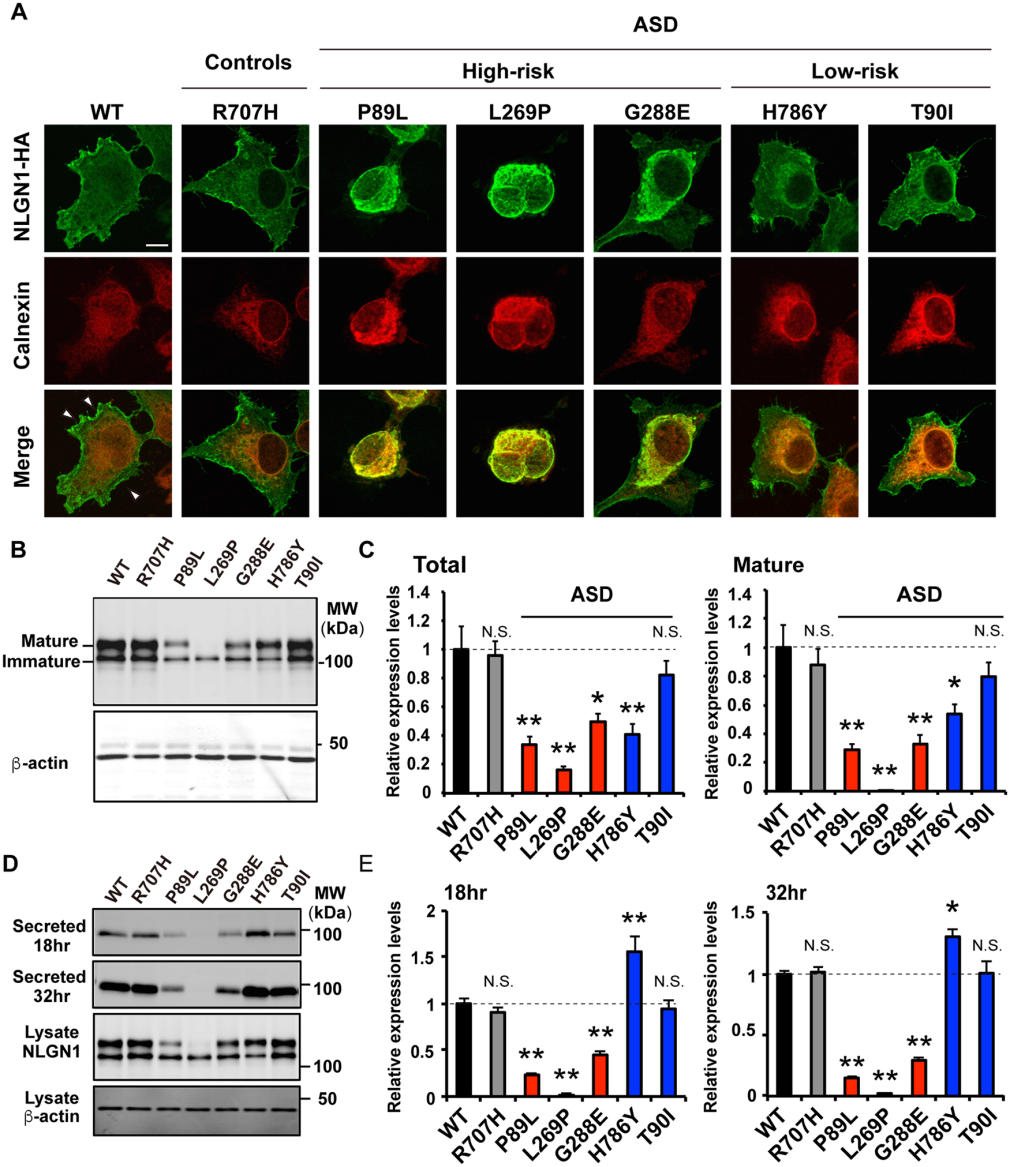
Pathogenic *NLGN1* variants exhibit abnormal sub-localization and expression. (A) Fluorescence images of COS7 cells transfected WT or mutant NLGN1 with HA-tag. Endoplasmic reticulum (ER) was stained with calnexin. Three pathogenic variants (P89L, L269P, and G288E) were trapped in ER, and failed to traffic the plasma membrane. Scale bar indicates 10 μm. (B) Representative images of western blots of cell lysates from COS7 cells transfected with HA-tagged NLGN1. NLGN1 was detected by anti-HA tag. The expected molecular weight for the NLGN1: glycosylated mature NLGN1 (~110 kDa), non-glycosylated immature NLGN1 (~100 kDa). (C) Quantitative analysis of the western blots for total (left) and glycosylated (right) NLGN1. The expression of NLGN1 variants is normalized to the corresponding β-actin. NLGN1 variants observed in ASD subjects showed decreased expression level compared to WT. Data represents mean ± S.E.M. of four samples from three independent experiments (one-way ANOVA followed by Tukey-Kramer’s multiple comparisons test, *p<0.05, **p<0.01 compared with WT). Gray, red, and blue bars indicate the non-pathogenic variant, “high-risk”, and “low-risk” pathogenic variants, respectively. (D) Representative image of western blots of conditioned media (collected at 18 and 32 hr after transfection), cell lysates (collected at 32 hr after transfection), and β-actin as an internal control. Both cleaved NLGN1 in media and full-length NLGN1 in cell lysate were detected by anti-HA tag. (E) Quantification of cleaved NLGN1 with the HA-tag inserted in extracellular domain in conditioned media. Relative expression levels at 18 hr post-transfection (left) and 32 hr post-transfection (right) are shown. Cleaved NLGN1 was decreased in three pathogenic variants (P89L, L269P, and G288E) and increased in H786Y variant compared to WT. Data represents mean ± S.E.M. of six samples (one-way ANOVA followed by Tukey-Kramer’s multiple comparisons test, *p<0.05, **p<0.01 compared with WT).

To investigate the impact of *NLGN1* variants detected in ASD subjects, we generated five constructs of mutant *NLGN1*, including three high-risk variants (mouse P89L, L269P, and G288E, which corresponds to human P89L, L269P, and G297E, respectively) and two low-risk variants (mouse H786Y and T90I, which corresponds to human H795Y and T90I, respectively). To compare the effect of variants found in ASD patients with that of controls, we also generated a construct of the non-pathogenic variant R707H (corresponding to human R716H, rs118079207 from dbSNP) that had been identified only in healthy control subjects. First, we examined subcellular localization of NLGN1 variants in COS7 cells. Consistent with the role of NLGN1 as a synaptic cell-adhesion molecule, NLGN1 wild-type (WT) was predominantly expressed at the plasma membrane (Fig 3A, S2 Fig). However, three NLGN1 high-risk variants (P89L, L269P, and G288E) were colocalized with calnexin and were not appropriately delivered to the plasma membrane, indicating that these construct-encoded mutant molecules are exclusively localized in endoplasmic reticulum (ER) likely due to misfolding of the protein by amino-acid substitution. In particular, P89L and L269P exhibited severe phenotypes. Conversely, two low-risk variants, H786Y and T90I, and the non-pathogenic variant, R707H, were not associated with changes in subcellular localization. The localization of these variants was further confirmed using the antibody against NLGN1 (S3 Fig).

We next examined the effect of the identified sequence variants on the NLGN1 protein level. In transfected COS7 cells, we observed a significant reduction of NLGN1 expression in four of five NLGN1 variants detected in ASD subjects, including three high-risk variants (P89L, L269P, and G288E) and a low-risk variant (H786Y). The protein level of the T90I variant was comparable with WT (Fig 3B and 3C). Three high-risk variants (P89L, L269P, and G288E) decreased the NLGN1 protein level likely through the ER-associated degradation and protein destabilization as previously described [24, 46, 47]. However, the H786Y variant showed significantly reduced expression without any abnormal cellular localization, indicating a different process of protein reduction. We assessed another possible mechanism by which the point mutation may induce NLGN1 degradation. Ectodomain shedding, which triggers the rapid degradation of NLGN1 as a mechanism to downregulate excitatory synapse activity, was reported [48, 49]. In this process, the extracellular domain of NLGN1 on the plasma membrane is cleaved and secreted to extracellular space. To determine whether this process is affected by H786Y, we quantified the cleaved NLGN1 in conditioned media from COS7 cells expressing NLGN1 variants. Quantification revealed that the amount of cleaved NLGN1 in the H786Y variant was increased at both 18 and 32 hour post-transfection compared to WT, suggesting excessive cleavage and degradation of NLGN1 in this variant (Fig 3D and 3E). However, the three high-risk variants exhibited the decreased amount of cleaved NLGN1, which is consistent with the localization in the ER and lack of plasma membrane expression of these variants. These results suggest that *NLGN1* variants found in ASD subjects (P89L, T90I, L269P, G297E, and H795Y) affected at least two types of degradation processes of NLGN1, ER-associated degradation and ectodomain cleavage, that led to the decreased expression of NLGN1.

**Fig 4:**
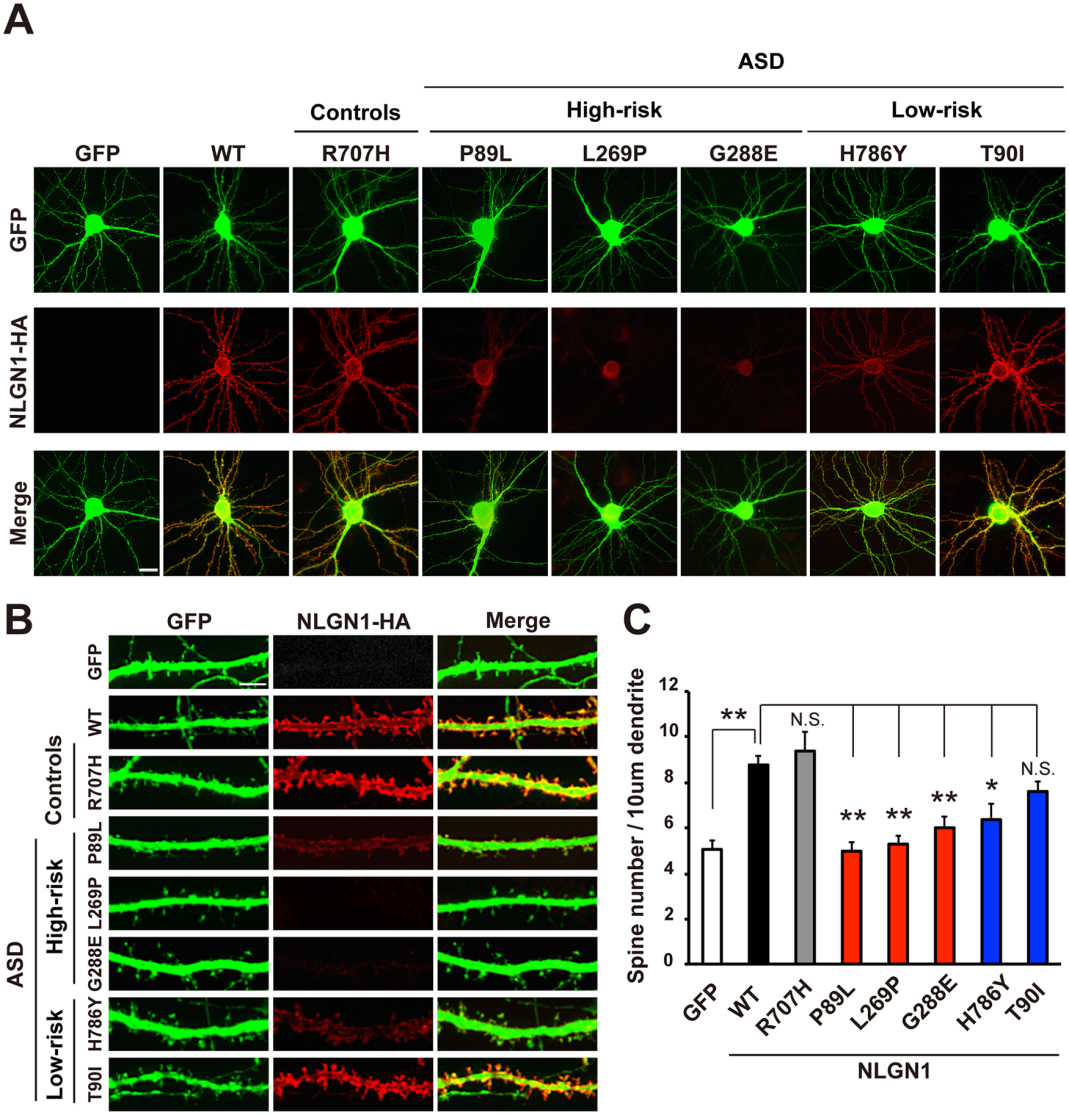
Impaired spine induction by *NLGN1* variants observed in patients with ASD. (A) Representative fluorescence images of hippocampal neurons (DIV14) co-transfected with negative control, WT, and non-pathogenic NLGN1 or pathogenic NLGN1 variants with GFP expression vector. NLGN1 expression of pathogenic variants (P89L, L269P, G288E, and H786Y) was decreased compared to others. (B) Representative images of spines from neurons transfected with negative control, WT, and non-pathogenic NLGN1 or pathogenic variants of NLGN1. (C) Quantification of the number of spines. Spine number in dendrite was significantly lower in four pathogenic variants (P89L, L269P, G288E, and H786Y) compared to WT, and no significant differences between GFP-transfected control and these four variants were observed. Data represent mean ± S.E.M. (*p<0.05, **p<0.01 Tukey-Kramer’s multiple comparisons test). More than 14 neurons are counted for each NLGN1 variant. Scale bar indicates 20 μm for (A) and 5 μm for (B).

To investigate whether decreased expression of pathogenic variants is also observed in neurons, we co-transfected NLGN1 variants with a GFP expression vector in primary hippocampal neurons. In WT as well as the non-pathogenic R707H and pathogenic T90I variants, robust expression of NLGN1 was observed in soma, dendrites, and spines (Fig 4A). In contrast, four pathogenic variants (P89L, L269P, G288E, and H786Y) showed weak expression of NLGN1 in neurons, which was consistent with the results in COS7 cells. Three high-risk variants tended to express only in soma and proximal dendrites, and weak signals were detected in spines. Overall, these results suggest that majority of *NLGN1* variants (four of five) identified in ASD subjects lead to reduced NLGN1 expression in both cell line and primary neurons.

### Functional impairment of *NLGN1* variants observed in patients with ASD

We then tested whether the reduction of NLGN1 affects dendritic spine formation. Quantification of spine numbers in NLGN1 variants transfected in hippocampal neurons revealed that WT, as well as the control R707H variant, had significantly higher spine numbers when compared with GFP transfected cells (Fig 4B and 4C). Similarly, T90I also increased spine numbers compared with GFP transfected cells to a similar extent as WT, suggesting its normal function of spine induction. In contrast, the number of spines in proteins harboring pathogenic variants (P89L, L269P, G288E, and H786Y) was significantly lower than that of WT. Thus, four pathogenic NLGN1 variants impaired spine formation, suggesting that the reduced protein level combined with changes in cellular localization may lead to pathogenesis.

### Generation of *Nlgn1* P89L KI mice

**Fig 5:**
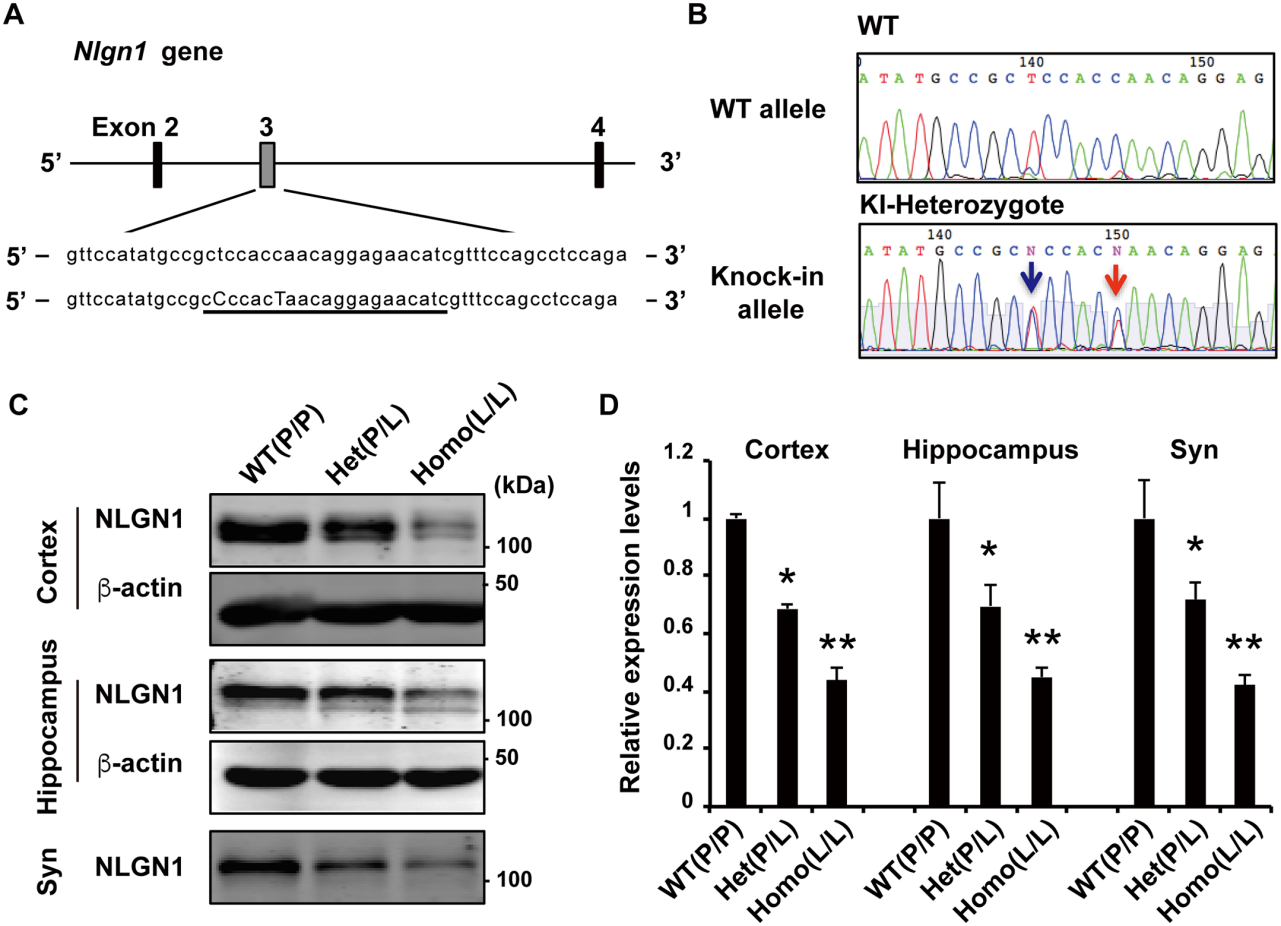
Generation of *Nlgn1* P89L KI mice. (A) Schematic of the mouse genomic locus of *Nlgn1* showing the target site of Cas9. sgRNA sequence is underlined, and replaced bases in knock-in mice are capitalized. (B) DNA sequence electropherograms of WT and knock-in heterozygote mouse. Red arrow indicates the amino acid substitution from proline to leucine (CCA to CTA of residue 89), and blue arrow indicates a silent mutation for genotyping using restriction enzyme *Bsr*BI. (C-D) Western blots of cortex, hippocampus, and cortical synaptosomal fractions from wild-type, *Nlgn1* P89L heterozygote, and homozygote mutant mice. (WT n = 3, heterozygote n = 4, homozygote n = 3) Data are represented as means ± S.E.M. (*p<0.05, **p<0.01 Tukey-Kramer’s multiple comparisons test).

Although the *NLGN1* variants found in ASD subjects displayed significant effects *in vitro*, we sought to investigate the effect of these variants at the organismal level. It remained to be established whether these variants lead to anomalies in ASD traits *in vivo*. We selected the P89L variant in *NLGN1* identified in this study for further analysis for the following reasons: 1) We initially identified this as a novel variant in two ASD siblings and absent in their unaffected sibling; 2) A mother who transmitted the variant to two ASD children exhibited psychiatric disturbances; 3) Significant phenotypes were observed *in vitro*. Thus, we generated KI mice with P89L substitution in *Nlgn1* as a possible novel mouse model for ASD. We used CRISPR/Cas9 system to incorporate the P89L substitution, along with a silent mutation at nucleotide 261 to delete a *Bsr*BI restriction enzyme site to facilitate genotyping [50] (Fig 5A and 5B). Fifteen off-target candidates were analyzed by direct sequencing of founder mice, and no off-target mutations have been detected in this system [51] (S2 Table).

Both homozygous and heterozygous *Nlgn1* P89L mutant mice were fertile and exhibited no gross abnormalities. No decrease of the *Nlgn1* mRNA was observed in mutant mice, but quantification of NLGN1 protein revealed reduced NLGN1 in the KI mouse brain, including cortex, hippocampus, and the cortical synaptosomal fraction, indicating that the P89L substitution destabilized the NLGN1 protein, consistent with *in vitro* observations (S4 Fig, Fig 5C). Overall, approximately a 30% reduction of NLGN1 expression in KI heterozygotes and a 60% reduction in KI homozygotes were observed (Fig 5D).

To examine the spine phenotypes, we counted the spine number in a primary hippocampal culture derived from *Nlgn1* P89L heterozygotes (P/L) and homozygote (L/L) as well as WT (P/P); however, no significant difference in the spine number was observed among three genotypes (S5A and S5B Fig). We further crossed these mice with *Thy1-YFP* line H transgenic mice and examined the spine number in the hippocampus CA1 slice. The result also showed no significant difference among three genotypes (S5C and S5D Fig).

### *Nlgn1* P89L mice showed abnormal social behavior and impaired spatial memory

In all ASD patients reported in our study, the NLGN1 variants, including P89L, were in a heterozygous state, suggesting haploinsufficiency and/or a gain-of-function effect underlying pathogenicity. Therefore to assess the behavioral effect of *Nlgn1* variant, we initially performed comprehensive behavioral tests of heterozygous mutants (S3 Table).

**Fig 6:**
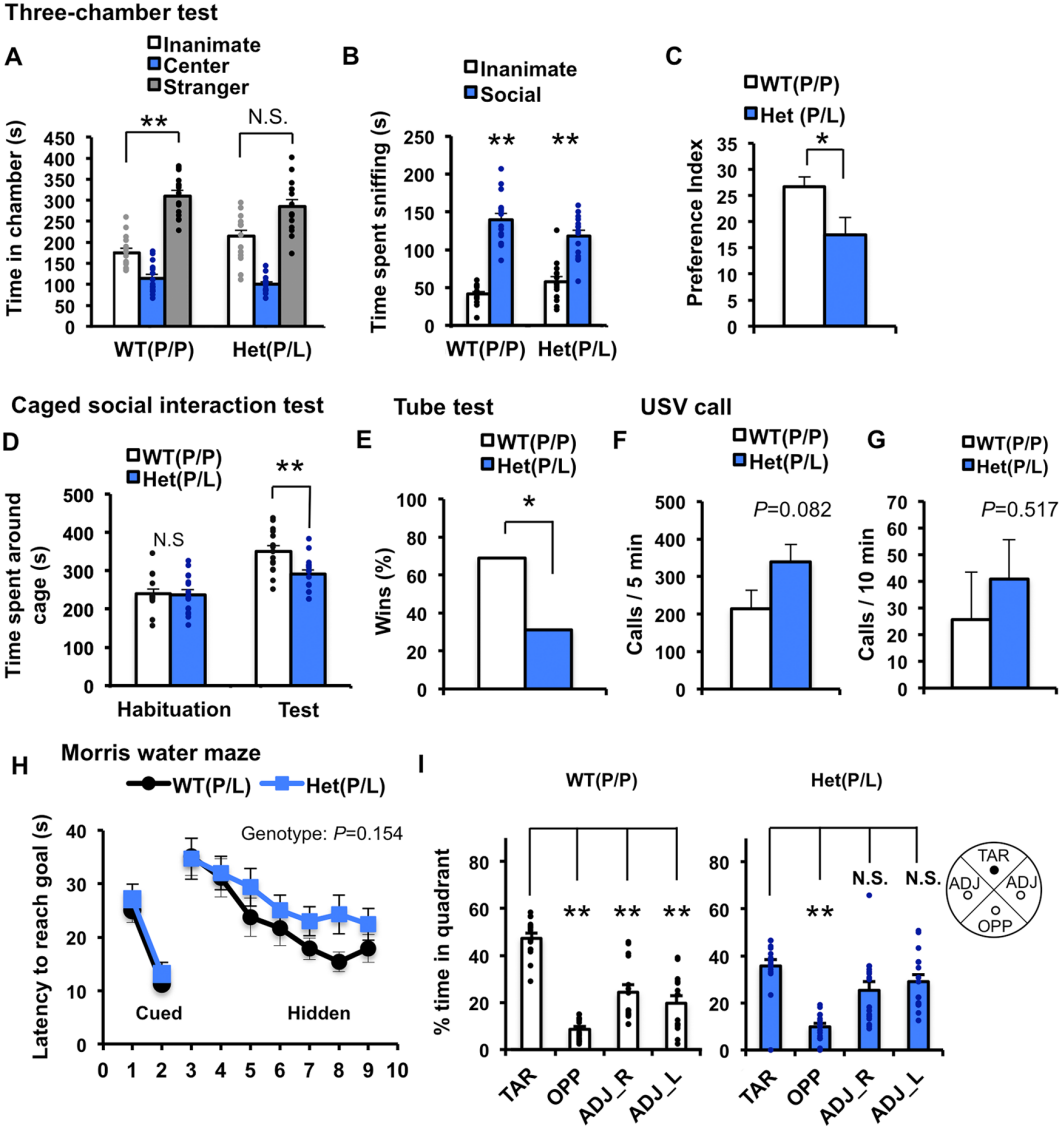
Comprehensive behavioral analysis of *Nlgn1* P89L mice. (A-C) Three-chamber social interaction test. (A) A stranger mouse was placed in one of the side chambers in a wired cage, and an empty wired cage was placed in the opposite chamber. Time spent in each chamber. (B) Time spent sniffing the stranger or the inanimate object. Both WT and *Nlgn1* P89L (P/L) mice spent more time sniffing the stranger than the empty cage. (C) Social preference index was lower in *Nlgn1* P89L (P/L) mice. Preference index = ((Time sniffing the stranger/ (Time sniffing the stranger + Time sniffing the inanimate)) x 100) - 50. *p<0.05, **p<0.01. Two-way repeated measures ANOVA, Bonferroni post-test (A, B). t-test (C), n = 15 for WT, n = 15 for heterozygous mutant. (D) Caged social interaction test in the open field. The test consists of two sessions, a 10 min habituation, followed by a 10-min test. Time spent around the cage during the habituation phase with an inanimate cage was identical between genotypes, however, time spent around the cage with an age-matched unfamiliar male mouse in the test session was significantly less in *Nlgn1* P89L hetrozygote (P/L) mice compared to WT. **p<0.01. t-test, n = 15 for WT, n = 16 for heterozygous mutant. (E) Wins frequency in the social dominance tube test. *Nlgn1* P89L (P/L) heterozygote mice had a lower winning rate. *p<0.05. Chi-square test. (F) The number of ultrasonic vocalizations emissions at postnatal day 7 induced by maternal-separation during 5 min session. (G) The number of ultrasonic vocalizations emissions from adult male mice during 10 min session. Adult female mouse was presented as stimuli. (H, I) Morris water maze to assess hippocampal-dependent spatial learning and memory. On day 1 and 2, mice were trained to find a visible platform in the water maze. On day 3 to 9, mice were trained to find a hidden platform. On day 10, spatial memory was assessed with the platform removed as a probe test (H) Quantification of latency to reach goal across days of training session from day 1 to 9. Two-way repeated measures ANOVA. (I) Quantification of time spent in each quadrant in a probe test session at day 10. TAR, ADJ and OPP indicates the target, adjacent, and opposite quadrant, respectively. WT showed significant preference for the target quadrant than the other quadrants, whereas *Nlgn1* P89L (P/L) mice do not show the preference for the target quadrant compared to adjacent quadrants. **p<0.001, t-test. n = 15 for WT, n = 16 for heterozygous mutant. Data are represented as means ± S.E.M. See S3 Table for all statistics.

To test whether *Nlgn1* P89L heterozygotes (P/L) show abnormal social behavior, we performed two types of social interaction tests, three-chamber [52–55] and caged social interaction tests [25, 56, 57]. In the three-chamber social interaction test, mice are given the choice to interact with either the inanimate object (an empty cage) or a cage containing a stranger age-matched male mouse, and the amount of social interaction behavior was measured. The significant effect of chamber was observed in both genotypes (Two-way repeated measures ANOVA, genotype p>0.05, stimulus p<0.01, genotype × stimulus p<0.1). Post-test revealed that WT showed a significant preference for the chamber with the stranger compared to the chamber with the inanimate object, but heterozygote mutant did not show a significant difference from WT mice (Fig 6A). Both genotypes spent more time staying around (S3 Table) or sniffing the cage with the stranger than the inanimate object (Fig 6B). Interaction between genotype and stimulus was significant (Two-way repeated measures ANOVA, genotype p>0.05, stimulus p<0.01, genotype × stimulus p<0.05) (S3 Table). We also compared the social preference index to evaluate additional parameters [28, 58–61], and *Nlgn1* P89L heterozygotes were significantly lower than WT (Fig 6C, S3 Table). These observations indicated not the robust social phenotype in *Nlgn1* P89L heterozygotes but potential impairment in social behavior. In the caged social interaction test, we observed mouse behavior in two sequential test phases. First, a mouse was allowed to explore the field with an empty cage (habituation phase), and then allowed to explore the field with the cage containing an age-matched adult male mouse (test phase). In this test, no difference was observed in the habituation phase between genotypes. However, *Nlgn1* P89L heterozygotes exhibited reduced interaction time with a stranger mouse when compared to WT suggesting the impaired social interaction (Fig 6D). As an additional test to evaluate social interaction, we performed the tube test for social dominance [62]. Mouse models of syndromic or non-syndromic ASD show altered behavior in this task [55, 62, 63]. In this test, two mice are placed in opposite ends of a clear and narrow tube, and interact with each other in the middle of the tube. A dominant mouse generally forces its opponent out of the tube. When a pair of WT mouse and *Nlgn1* P89L heterozygote was matched, *Nlgn1* P89L heterozygotes more often retreated from WT mice (Fig 6E). These findings imply that *Nlgn1* P89L heterozygote mice have impaired social interaction behavior compared to WT. Additionally, we examined the olfactory habituation/dishabituation and novel object recognition tests of mutant mice, but no difference was observed between genotypes in both tests. Thus odor discrimination and object recognition of mutant mice were not affected (S6 Fig).

We also analyzed ultrasonic vocalizations (USVs) of mutant mice at both postnatal day 7 (P7) (Fig 6F) and the adult stage (four-month old) (Fig 6G). At both ages, we did not observe a significant difference in call numbers (S3 Table). There was also no significant difference in repetitive behavior between genotypes assessed by counting time spent for grooming and the marble burying test, general locomotor activity assessed by the open field test, anxiety-like behavior assessed by the elevated plus maze task, and acoustic startle reactivity by the acoustic startle response test (S3 Table).

Previous studies revealed that *NLGN1* is involved in memory function [56, 64, 65]. In fact, several ASD subjects with *NLGN1* variants reported in this study exhibited lower IQ (L269P: IQ = 40; G297E: IQ = 79). To test spatial memory of mutant mice, we performed the Morris water maze test. During the initial training period, *Nlgn1* P89L heterozygotes were slightly delayed to reach the target, but no significant difference was observed between genotypes in latency and distance traveled to reach the platform (Fig 6H, S3 Table). However, in a probe trial, *Nlgn1* P89L heterozygotes spent significantly less time in the target quadrant compared with WT (S3 Table). Moreover, although WT mice showed a significant preference for the target quadrant, and spent significantly more time in the target quadrant than the other quadrants, heterozygotes did not exhibit a difference between time in target and adjacent quadrants (Fig 6I). These results suggest that spatial memory, in addition to social interaction, is affected in *Nlgn1* P89L heterozygote mice.

To examine whether additional decrease in *NLGN1* level leads to a more severe phenotype, we performed behavioral tests on *Nlgn1* P89L homozygotes (L/L) and compared their phenotypes to WT littermates. We did not observe the differences in general locomotor activity, repetitive behavior and anxiety-like behavior, similar to heterozygotes (S4 Table). To confirm the abnormal social behavior, we performed a three-chambered social interaction, a caged social interaction and tube test. Similar to *Nlgn1* P89L heterozygote mutants, homozygotes showed a significantly lower winning rate compared to WT littermates (S7E Fig). Contrary to our expectations, we observed the normal social behavior in homozygote mice both in the three-chambered social interaction and the caged social interaction tests (S7A–S7D Fig). The time spent in chambers, time spent around cages, social preference index were comparable to WT. We also examined USV calls at P7, and similar to heterozygous mutants, no difference was observed in call numbers (S7F Fig). To investigate the spatial memory of homozygote mice, we performed a Morris water maze test. During training phase, homozygote mutant mice showed a mild but a significant delay of learning both in distance traveled and latency to reach goal (S7G Fig). In a probe trial, WT mice showed a significant preference for the target quadrant, and spent more time in it compared to all other quadrants. However, homozygote mice exhibited no difference between time in target and opposite, and target and adjacent left quadrant (S7H Fig). Similar to heterozygous mice, homozygotes also showed a modest deficiency in spatial memory. Although the behavioral phenotypes of *Nlgn1* P89L homozygotes showed similar abnormalities to heterozygotes, they displayed milder phenotypes in the social interaction assays compared to heterozygous mice.

## Discussion

Components of the trans-synaptic complex, including neurexins and neuroligins, represent one of the most intensively studied pathways in ASD research [9, 10]. Nonetheless, to date, none of the *NLGN1* variants detected in patients with ASD have been systematically analyzed. In the present study, we examined the role of pathogenic *NLGN1* variants both *in vitro* and *in vivo*. Notably, mutant mice with a novel variant P89L in *Nlgn1* displayed both cellular and behavioral abnormalities that may underlie pathophysiology of ASD.

First, we identified four variants (P89L, T90I, L269P, and G297E) in the extracellular and one (H795Y) in the intracellular domain of *NLGN1* as putative pathogenic variants observed in ASD subjects. Structurally, NLGNs belong to the α/β-hydrolase fold superfamily of proteins and have a large extracellular region consisting of the α/β- hydrolase fold domain (esterase-homology domain) [44]. Since it has been shown that variants in the α/β- hydrolase fold domain often influence protein folding and trafficking [24, 44, 46, 47], three pathogenic *NLGN1* variants localized in this domain (P89L, L269P, and G297E) may impair proper protein folding and trafficking to the plasma membrane. However, the functional consequence of variants in the intracellular domain of NLGNs is still largely unknown. In this study, we also provide evidence for the unique functional effect revealed by the analysis of the H786Y allele (corresponding to H795Y in human). The H786Y variant in the intracellular domain of *NLGN1* leads to excessive cleavage of *NLGN1*, which was concomitant with decreased *NLGN1* expression. This cleavage process may be linked to the activity-dependent degradation of NLGNs [48, 49] implicated in the regulation of synaptic remodeling and transmission. Although the specific biological and functional significance of this process remains unknown, the H786Y variant caused decreased expression of *NLGN1* protein in the cells and impaired induction of dendritic spines. Collectively, our analyses indicate that several cellular mechanisms, combining reduced protein level and abnormal cellular localization may underlie pathogenic effects of *NLGN1* variants, leading to impaired neuronal function.

Second, we asked whether a newly identified variant in *NLGN1* may lead to anomalies in ASD-associated behavior. A previous report showed that *Nlgn1* deletion mice presented relatively mild abnormalities in behavior, especially in social behavior [56]. Our human genetic study identified only heterozygous point mutations in the *NLGN1* gene. This is not surprising; the reported *NLGN1* variants are rare and therefore the likelihood of finding these variants in a homozygous or even compound heterozygote state is low. In addition to the rare variants found in ASD subjects (described in this paper), several rare predicted deleterious variants are listed in the ExAC database [66]. The subjects included in the ExAC collection (n = 60,000) cannot be considered as “healthy controls”. These subjects have been ascertained for biomedically important diseases, and the team excluded severe pediatric conditions [66]. We assume that a small number of carriers of deleterious *NLGN1* mutations may have milder behavioral anomalies and therefore would not be excluded from exome analysis. Functional analyses of *NLGN1* variants, such as those reported in our paper, may represent an appropriate justification for the re-contact of rare carriers, including the AGRE family sequenced in our study, and their deep-phenotyping.

To analyze the behavioral consequence of the *Nlgn1* heterozygous point mutation, we generated KI mice of P89L, which recapitulate mutations found in two brothers with ASD. Although the *Nlgn1* P89L heterozygous mice showed only a 30% reduction of *NLGN1* protein in their brain, they displayed aberrant behavior, such as deficits in social interaction, altered social dominance and impaired spatial memory, without affecting basal locomotor activity and anxiety. These findings indicate that even heterozygotes of a point mutation in *NLGN1* contribute to aberrant behavioral traits relevant to ASD.

The impairment of spatial memory and mild deficits in social interaction were previously described in *Nlgn1* KO mice [56]. Similarly, we observed these deficits in *Nlgn1* P89L mutants. To evaluate the phenotype of social interaction, we applied the three-chambered social interaction test in addition to the caged social interaction test which was used to test the *Nlgn1* KO mice [56]. The lower interaction time with the stranger mouse in the caged social interaction test was consistent with the result of *Nlgn1* KO mice. In the three-chambered social interaction test, the analysis of time spent in chamber showed the significant effect of the place of chamber by two-way repeated ANOVA, and the analysis of time spent sniffing revealed that both genotypes spent significantly more time interacting with the stranger mouse than the inanimate. However, another parameter or the further statistical analysis revealed the differences between genotypes. Two-way repeated ANOVA followed by post-test described in Veenstra-VanderWeele *et al*. [55] detected the effect of chamber place only in WT, not in *Nlgn1* P89L. The preference index calculated by time spent sniffing was significantly lower in *Nlgn1* P89L mice. These observations suggested a mild deficit in social interaction in *Nlgn1* P89L.

Electrophysiological studies on *Nlgn1* KO mice reveal that NLGN1 is required for long-term potentiation (LTP) in CA1 pyramidal neurons and modulates striatal glutamatergic neurotransmission [56, 67–69]. Alteration of the synaptic function may result in impaired spatial memory and increased repetitive behavior, respectively. Both *Nlgn1* KO mice and *Nlgn1* P89L KI mice demonstrate impaired spatial memory, however, *Nlgn1* KO mice display a strong grooming phenotype, whereas *Nlgn1* P89L heterozygotes did not show anomalies in repetitive behavior. Furthermore, unexpectedly, homozygotes show even a milder social behavior deficit and do not indicate more severe phenotypes when compared with heterozygotes. These correlations between gene expression levels and phonotypes are puzzling. Additional *in vivo* and *in vitro* experiments will be needed to resolve the molecular mechanisms underlying the effect of the NLGN1-P89L allele. Although our study showed a reduced protein level from the mutant and wild-type allele, we cannot exclude a gain-of-function effect of the NLGN1-P89L allele, combined with the dosage effect. Another puzzling finding relates to the effect of the NLGN1-P89L mutation on synaptic structure. While *Nlgn1* KO mice show no reduction in synapse number *in vivo* [56], *Nlgn1* P89L heterozygotes display a significant reduction in synapse number *in vitro* but no reduction *in vivo*. This discrepancy might reflect that synapse number is insensitive to absolute NLGN1 levels but depends on transcellular differences in the relative amounts of NLGN1 [70]. The difficulty in comparing our findings on the NLGN1-P89L allele with the published data on behavioral phenotypes of the *Nlgn1* KO represents a limitation of our study. However, future studies that combine a comparison of genetic, biochemical and electrophysiological findings on several *NLGN1* alleles in parallel will resolve complexities of synaptic anomalies and their link to human disease.

Recent genomic studies identified CNVs of *NLGN1* in ASD [71–74]. With respect to ASD etiology, a common copy number duplication in the non-coding region of *NLGN1* seems to increase the risk of ASD pathogenesis, although the impact of these variants on NLGN1 protein is unclear [71]. Moreover, altered *NLGN1* levels in brain were observed in multiple lines of ASD model mice such as *Eif4ebp2* knockout and *Fmr1* knockout [29, 30]. Of note, adjusting the NLGN1 level in these mice rescues ASD phenotypes such as social impairment. Thus, the proper level of *NLGN1* may be indispensable for brain development and maintenance of neuronal network. Similarly, dosage sensitivity has been reported for several genes contributing to the risk for a range of psychiatric disorders, including Rett Syndrome [75]. It is striking that the AGRE database includes information on psychiatric disturbances in the mother and several relatives of two ASD siblings with the P89L variants (mother’s mother with panic attacks, mother’s cousin with mild developmental delay, and mother’s nephew with ADHD). Although we provide strong evidence for the role of *NLGN1* in synaptic spine formation (*in vitro*) and in social behavior in mice, the relevance of these rare variants to ASD will require further genetic evaluation in a larger number of ASD subjects. ASD is a highly polygenic disorder and a cumulative effect of a number of pathogenic variants with a small and intermediate effect leads to the disease [7, 36]. Our analysis of exome data for two ASD siblings who share *NLGN1* variants (P89L) can serve as an example for this complexity, because in addition to this variant, each child shared novel deleterious variants in 6 additional genes (*RFX1*, *PPP2R5C*, *C2CD5*, *NCAM2*, *ZBTB1*, and *DISP1*). Interestingly, mouse orthologs of three of these genes (*RFX1*, *PPP2R5C*, and *DISP1*) are essential (lead to embryo-lethality in homozygotes) and therefore likely to add to the cumulative effect on the ASD susceptibility [76].

In summary, our comprehensive cellular analyses of *NLGN1* variants and behavioral deficits in a novel *Nlgn1*-P89L mutant mouse provide the first evidence that *NLGN1* variants found in human patients are directly involved in ASD-like phenotypes through multiple mechanisms. Our study reinforces the critical association between genetic variants in *NLGN*s and neuropsychiatric disorders.

## Materials and methods

### Clinical features of patients (AU072904, AU072905)

A pair of siblings affected with a non-syndromic autism, AU072904 and AU072905 from the Autism Genetic Resource Exchange (AGRE) was evaluated. Their parents first became concerned about autistic features in their child (AU072904) when he was 0–12 months old and in their second child (AU072905) when he was 12–24 month old. Both siblings with ASD presented severe deficits in verbal and nonverbal communication, abnormal stereotypies, and abnormal sensory interest. AU072904 had more severe symptoms and was also diagnosed with ADHD and OCD when he was 3-year-old and 4-year-old, respectively. AU072905 showed OCD-like symptoms as well, but had not been diagnosed. Motor function was normal in both siblings.

### DNA constructs

The plasmid expressing Hemagglutinin (HA)-tagged mouse NLGN1 was obtained from Addgene (#15260). The constructs with NLGN1 variants were derived by site-directed mutagenesis using the following primers: P89L (F: 5’- GCCGCTCCACTAACAGGAGAAC -3’, R: 5’- GTTCTCCTGTTAGTGGAGCGGC 3’), T90I (F: 5’- TGCCGCTCCACCAATAGGAGAACATCG -3’, R: 5’- CGATGTTCTCCTATTGGTGGAGCGGCA -3’), L269P (F: 5’- GTTCATGTGTCAACCCGCTGACTTTATCC -3’, R: 5’- GGATAAAGTCAGC GGGTTGACACATGAAC -3’), G288E (F: 5’- CAATAGCTCAGAGTGAAACAGCCCTTTCC -3’, R: 5’- GGAAAGGGCTGTTTCACTCTGAGCTATTG -3’), H786Y (F: 5’- GATTCAGCCCTTATATACATTCAACAC -3’, R: 5’- GTGTTGAATGTATATAAGGGCTGAATC -3’), and R707H (F: 5’- CAGCCCT CAGCACACGACCACCAAC -3’, R: 5’- GTTGGTGGTCGTGTGCTGAGGGCTG -3’). Mutated DNA was identified by *Dpn*I selection for hemimethylated DNA, and the sequence of each clone was verified by DNA sequencing.

### Cell culture and transfection

COS7 cells were maintained in DMEM media, supplemented with 10% fetal bovine serum. Cells were seeded on 12 well plates and transfected with lipofectamine 2000 or lipofectamine 3000 (Invitrogen), according to the manufacturer's protocol. The equal amount of NLGN1 construct was transfected in the amount of 1.3 μg per well for Western blots and 400–800 ng per well for immunofluorescence image. Assays were conducted 24–48 hours after transfection.

### Western blot analysis

Cortex and hippocampus of mice were rapidly dissected and snap-frozen in liquid nitrogen. The tissues were homogenized in ice-cold TNE buffer (20 mM Tris-HCl, pH 7.4, 150 mM NaCl, 2 mM EDTA, 1% NP-40), and centrifuged at 15,000 × *g* for 20 min at 4°C. The protein concentration was determined using the BCA protein assay (SIGMA). Cortical synaptosomes were prepared as previously described [77]. Briefly, the dissected cortex was homogenized in ice-cold HEPES-buffer (4 mM HEPES pH 7.4, 0.32 M sucrose), and centrifuged at 1,000 g for 15 min. The supernatants were subsequently centrifuged at 10,000 g for 15 min to yield the crude synaptosomal pellet. The resulting pellets were lysed with TNE buffer. Immunoreactive bands were visualized by ODYSSEY Infrared Imaging System (LI-COR), and the intensity of the band was calculated by the same system. Goat polyclonal antibody against NLGN1 (Santa Cruz) and mouse monoclonal antibody against β-actin (Sigma) were used. To detect transfected HA-tagged NLGN1 variants in cultured cells, rabbit monoclonal antibody against HA (Cell signaling) was used. Rabbit anti-NLGN1 antibody used in S3 Fig is a generous gift from Dr. Peter Scheiffele (University of Basel).

### Immunofluorescence

COS7 cells seeded on coverslips were fixed with methanol at -20°C for 40 min, and then blocked with 5% Normal Goat Serum. HA-tagged NLGN1 was detected by mouse monoclonal antibody against HA (Cell Signaling), and ER was stained with rabbit monoclonal antibody against calnexin (Enzo). Primary neurons were fixed with 2% PFA, 4% sucrose / PBS. Rabbit monoclonal antibody against HA (Cell Signaling) and mouse monoclonal antibody against GFP (Thermo) were used. The coverslips were mounted on Vectashield mounting medium (Vector Lab). Confocal images were obtained using the laser scanning confocal microscopy (FV-1000, Olympus).

### Imaging and quantitative analysis of spine density

Primary cultures of hippocampal neurons from embryonic day 16.5 mice (ICR) or P0 mice (*Nlgn1* P89L mutants or their WT littermates) were prepared and cultured as described elsewhere [78] with some modifications. Cells were plated on poly-l-lysine coated coverslips in 12 well palates. Equal amounts of each construct, 150 ng NLGN1 and 450 ng GFP, were co-transfected at day *in vitro* (DIV) 7–8, and cells were fixed at DIV 14–15. Density and morphology of dendritic spines were analyzed by laser scanning confocal microscopy (FV-1000, Olympus). Progeny from a cross between the *Nlgn1* P89L mutant mice and *Thy1*-YFP line H transgenic line [79] were used to label hippocampal neurons. Fifty-μm-thick coronal sections were obtained by vibratome (Leica, VT1200S). Spines in apical dendrites of hippocampus CA1 were quantified.

### Whole exome sequence (WES)

AU072904 (4, Fig 1A) and AU072905 (5, Fig 1A) from a multiplex family (AU0729) from the Autism Genetic Resource Exchange (AGRE) [33] were evaluated. The exomes were captured with the Agilent SureSelect Human All Exon kit and paired-end sequencing (2 × 100) was performed with Illumina HiSeq 2000 sequencer according to the recommended protocols form the manufacturers. The short reads were aligned to hg18 human genome with Burrows-Wheeler Aligner (BWA) (0.5.9rc1) [80] and variant calling was performed with the Genome Analysis Toolkit (GATK) (1.0.3116) [81] with default protocols. For AU072904 and AU072905, respectively, we identified 23,614 and 23,329 single nucleotide variants (SNVs) that passed the GATK valiant quality score recalibration (VQSR) filter. The mean depths of coverage for called variants that passed the VQSR filter were 98.76 and 92.78, respectively. *NLGN1* (T90I) was identified among 362 ASD children recruited at Children's Hospital of Philadelphia with exonic variants captured by Illumina HumanExome-12 BeadChip.

### Structure modeling

The structure model was drawn using the UCSF Chimera package [82] (https://www.cgl.ucsf.edu/chimera/). The free energy changes of NLGN1 were calculated using FoldX (version 4.0, http://foldxsuite.crg.eu). The calculation was performed three times.

### CRISPR/Cas plasmid

*Nlgn1* sgRNA was determined by the CRIPSR Design tool (http://crispr.mit.edu/). The 20-bp guide sequence, 5’- GATGTTCTCCTGTTGGTGGAG -3’, was cloned into pX330 vector (Addgene #42230). The cleavage efficiency of sgRNA was confirmed by surveyor assay using Surveyor mutation detecting kit (Transgenomic).

### Generating P89L KI mice

*Nlgn1* sgRNA and Cas9 mRNA were transcribed using MEGAshortscript T7 Kit (Thermo) and mMESSAGE mMACHINE T7 Ultra Kit (Thermo), respectively. RNAs were purified using MEGAclear kit (Thermo). The donor single-stranded oligonucleotide containing target mutations was synthesized and purified by PAGE (Sigma). The target sgRNA (25 ng/ul), Cas9 mRNA (50 ng/ul), and the donor oligonucleotide (25 ng/ul) were microinjected into the cytoplasm of fertilized eggs collected from C57BL/6J mice.

### Genotyping

Genotypes of the mutant mice were established by PCR amplification, followed by *Bsr*B1 restriction enzyme treatment. The following primers were used. F: 5’- GCATTCTACCACCATTCCATCTTTC -3’, R: 5’- TGGAAGAAAGAAAATGAGTAGGCACAG -3’. Genotyping of the founder mice was conducted by direct Sanger sequencing.

### Off-target analysis

Candidates for off-targets were selected by CRISPR design tool scoring of the off-target risk (http://crispr.mit.edu/). Potential off-targets were verified by direct sequencing of genomic DNA of founder mice.

### Animals

All procedures for animal experiments were carried out in accordance with the guidelines of the Animal Experimentation Committee of RIKEN Brain Science Institute. Mice were kept in 12 h light/dark cycle (light on at 8:00) with ad libitum access to water and food. All behavioral experiments were performed during light phase. The offspring of mating pairs were weaned on postnatal day 21, genotyped, and housed in sex-matched groups in standard mouse cages in accordance with the RIKEN Animal Care and Use Committee.

### Behavioral analysis

Comprehensive behavioral analysis was performed in male mice except for pup’s USV test. Both male and female were used in the recording of pup’s USV. 15 WT and 16 *Nlgn1* P89L heterozygote mice were used for the self-grooming test at the age of 11-week old, open field test (12-week old), marble burying test (13-week old), caged social interaction test (14-week old), USV test (15-week old), acoustic startle response test (15- to 16-week old) and Morris water maze (17- to 20-week old) in the described order. Additionally, 15 mice of each genotype at the age of 15-week old were used for the three-chambered social interaction test. For the elevated plus maze test, 15 WT and 20 *Nlgn1* heterozygote mice at the age of 15- to 16-week old were used. Finally, 15 WT and 15 *Nlgn1* heterozygote mice at the age of 12-week old were used for the tube test. Mice were acclimated to the test room for at least 30 min prior to testing, except for pup’s USV recording. In the pup’s USV recording, mice were acclimated for at least 1 hr prior to the testing.

### Open field test

Locomotor activity was measured by the open field test. Mice were placed in the open field apparatus (50 × 50 cm^2^) facing the wall, and their spontaneous behaviors were monitored for 60 min. Horizontal locomotor activity and time spent in the center were automatically recorded. The arena was illuminated with 100 lux.

### Elevated plus maze test

The elevated plus-maze consisted of two open arms (25 × 5 cm) and two enclosed arms of the same size, with 15 cm high transparent walls. The arms and central square were made of white plastic plates and were elevated to a height of 55 cm above the floor. Mouse behavior was recorded during a 10-min test period. Anxiety was measured by the percentage of time spent in the open arms. The maze was illuminated with 100 lux.

### Caged social interaction test

The caged social interaction test was performed in a 60 × 60 cm^2^ arena illuminated to 20 lux. A small wire cage was placed with or without age-matched WT male mouse, allowing auditory, visual, olfactory, and minimal tactile interaction [25, 83]. To prevent climbing by the subject mouse, weights were placed on the top of cages. The test consists of a 10 min habituation phase, and following a 10 min test phase. In a habituation phase, each mouse was allowed to explore the arena in an empty wire cage freely. In the test phase, an age-matched unfamiliar C57BL/6J male was enclosed in the cage, and the subject mouse was allowed to interact with it. The arena was illuminated with 20 lux.

### Three-chambered social interaction test

The three-chambered social interaction test was performed as previously described [53]. The test arena is a rectangular three-chambered box. The arena was divided by two Plexiglas walls with small square openings (5 × 3 cm) allowing access into each chamber. Each chamber was 20 × 40 × 22 cm in size, and each of two side chambers contained a small wire cage. The arena was illuminated with 20 lux. Initially, mice were placed in the middle chamber and allowed to explore the environment freely for 10 min for habituation. After the habituation period, an age-matched unfamiliar C57BL/6J male (stranger) that had no prior contact with the subject mouse was placed in one of the wire cages. Then the subject mouse was first placed in the middle chamber and allowed to explore the entire social test box for a 10-min session. Positions of empty cages and cages containing the stranger were counterbalanced. In the third phase, another unfamiliar mouse was enclosed in the empty cage. The subject mouse was allowed to explore with a choice of the familiar stranger or a novel stranger for 10 min. The behavior was recorded by a video camera, and number of entries and the amount of time spent in quadrant around wire cage were measured automatically. Time spent sniffing was manually scored by an observer blind to genotype.

### Ultrasonic vocalization (USV)

Ultrasonic vocalizations were recorded from isolated pups or adult male mice. Total number of calls and categories of USVs were analyzed using R.

(1) Pups’ USV

Pups were tested on postnatal day-7. Each pup was removed from their mother and put into a plastic beaker placed in a dark soundproof box. To maintain the pup’s body temperature, absorbent cotton was placed in the beaker. USV was recorded with an ultrasound detectable microphone connected to a pre-amplifier (Avisoft Bioacoustics, Berlin, Germany) set at a 250 kHz sampling rate for 5 min.

(2) Adult male mice USV

Fifteen- to sixteen-week-old male mice were tested. Each mouse was individually put into a plastic cage placed in a dark soundproof box. Then a wild-type unfamiliar female mouse (10-week-old) was put into the same cage, and the subject male mouse was allowed to communicate with the female mouse for 10 min.

### Tube test

A 30 cm long, 3.2 cm diameter clear acrylic tube was used for the test. Each mouse was passed through the tube 3 times per day for 3 days, and habituated to the test tube before testing. The test room was illuminated at 105–115 lux. A pair of mice (one *Nlgn1*-P89L mutant and one WT mouse from different cages) was released simultaneously from the opposite end of the tube. Each mouse was tested three times, paired with different mice from different cages in each trial. The entry side (left or right) was counterbalanced. The test ended when one mouse completely retreated from the tube.

### Self-grooming test

Each mouse was placed individually into a clean transparent plastic cage. The behavior was recorded by a video camera for a 10 min test period following 10 min habituation. The amount of time the subject spent grooming was counted by stopwatch. The test room was illuminated at 105–115 lux. The light intensity in the test cage was ~80 lux.

### Marble burying test

Each mouse was individually placed in a transparent polycarbonate cage containing 5 cm bedding (ALPHA-dri) with 20 clear blue glass marbles. After 30 min, mice were removed from the cage and the number of buried marbles (>50% covered with bedding) was recorded. The light intensity inside the cage was ~55 lux.

### Acoustic startle response test

Each mouse was tested for acoustic startle reactivity. Mice were placed in a Plexiglas cylinder, and acclimated for 5 min. Acoustic stimuli were presented at 0, 80, 90, 100, 110, and 120 dB in a pseudo-random order. The average inter-trial interval was 15 seconds (range: 10–20 s). The average startle amplitude of six presentations was calculated. The background white noise level in each chamber was 65 dB.

### Morris water maze test

The Morris water maze task was conducted as previously described [53]. A circular maze was filled with the water (20–22°C), rendered opaque by nontoxic white paint. The platform was placed in one of the four quadrants and submerged 1 cm below the surface. The maze was illuminated with 100 lux. Each trial began by placing the mouse into one of the four quadrants facing the pool wall. Training trials lasted up to 60 seconds. A mouse that failed to reach the platform within 60 seconds was subsequently guided to the platform, and placed on it for 10 seconds. Mice were trained to find a platform with a visual cue on days 1–2 (five trials per day) to confirm their visual or motor ability. On days 3–9, hidden platform tests were conducted (four trials per day) to evaluate spatial memory with an inter-trial interval of 25–30 min. A probe test was conducted on day 10. Different order of start positions was applied every day, but the identical order of start positions was used for all subject mice. Data were collected by using a video tracking system, and latency to reach the platform, distance traveled to the platform, and average swim speed were automatically recorded.

### Novel object recognition

The novel object recognition test was conducted as previously described [84] with some modifications. Briefly, one day before the test, mice were habituated to an open field arena for 30 min. The test consists of three phases, a 10-min habituation phase, a 10-min familiarization phase and following a 5-min test phase. In a habituation phase, each mouse was allowed to explore an empty open field arena freely. In a familiarization phase, two identical objects were placed and the subject was allowed to explore it. One hour later, each mouse was placed back in the arena, and allowed to explore one familiar object and one novel object. The arena was illuminated with 20 lux. The behavior was recorded by a video camera, and the amount of time the subject spent sniffing the object was counted by stopwatch.

### Olfactory habituation/dishabituation

The olfactory habituation/dishabituation test was conducted as described previously [53] with some modifications. One hour before the test, each mouse was placed into an empty cage with a cotton ball for the acclimatizing of the cotton ball itself. The cotton ball was enclosed in a plastic mesh case adhered on the wall of the cage. Nonsocial (water, almond and banana) or social smells were presented sequentially. Each smell was presented for 2 min and then replaced by a new applicator, three times in succession for a total of 15 presentations. The behavior was recorded by a video camera, and the amount of time the subject spent sniffing the cotton ball was counted by stopwatch. The test room was illuminated with 105–115 lux. The light intensity in the test cage was ~80 lux.

### Statistics

Statistical analyses were performed with EZR, which is a graphical user interface in R (The R Foundation for Statistical Computing, Vienna, Austria) [85]. The R we used was a modified version of R commander designed to add statistical functions frequently used in biostatistics.

## Supporting information

S1 Fig NLGN1 expression in human brain. (A) NLGN1 expression in human brain from prenatal to postnatal stages according to Human Brain Transcriptome database (http://hbatlas.org/). Full line indicates birth. NCX: neocortex, HIP: hippocampus, AMY: amygdala, STR: striatum, MD: midbrain, CBC: cerebellar cortex. (B) Calculated result of free energy changes by FoldX. Calculation was performed three times independently. Data represents mean ± S.D.
(PDF)

S2 Fig No alteration in the cell shapes of transfected COS7 cells. Representative fluorescence images of COS7 cells transfected WT or mutant NLGN1 with HA-tag. GFP was co-transfected to visualize cell shape. Scale bar indicates 10 μm.
(PDF)

S3 Fig The sub-localization of NLGN1 variants by anti-HA antibody and anti-NLGN1 specific antibody. Representative fluorescence images of COS7 cells transfected WT or mutant NLGN1 with HA-tag. NLGN1 sub-localization was detected by anti-HA antibody or anti-NLGN1 antibody, respectively. The signals from two antibodies were almost perfectly merged. GFP was co-transfected to visualize the cell shape. Scale bar indicates 10 μm.
(PDF)

S4 Fig *Nlgn1* mRNA expression in the cortex. Relative *Nlgn1* mRNA expression in the cortex. n = 3 for WT (P/P), n = 5 for heterozygous mutant (P/L), n = 3 for homozygous mutant (L/L). Data represents mean ± S.E.M.
(PDF)

S5 Fig Spine number in *Nlgn1* P89L mutant mice. (A) Representative fluorescence images of primary hippocampal neurons (DIV14) collected by WT, *Nlgn1* P89L heterozygotes, and homozygote embryos. GFP was transfected to visualize the cell shape. (B) Quantification of the number of spines. No difference was observed among genotypes. n = 3 for WT, n = 5 for *Nlgn1* heterozygotes, n = 3 for *Nlgn1* homozygotes. (C) Representative images of apical dendrites in hippocampus CA1 from WT, *Nlgn1* P89L heterozygotes, and homozygote mice. WT or *Nlgn1* P89L mutant mice was crossed with *Thy1-YFP* line H transgenic strain, and hippocampal neurons were sparsely labeled by YFP. n = 3 for each genotype. (D) Quantification of the number of spines. No difference was observed. Data represent mean ± S.E.M. Scale bar indicates 5 μm.
(PDF)

S6 Fig Olfactory habituation/dishabituation test and novel object recognition test of *Nlgn1* P89L mice. (A) Olfactory habituation/dishabituation test. The olfactory habituation and dishabituation responses to sequential presentations of water, two nonsocial odors (Almond / Banana), and two social odors. *Nlgn1* P89L heterozygote (P/L) mice showed normal olfactory habituation and dishabituation responses. (B) Novel object recognition test. Both WT and *Nlgn1* P89L (P/L) mice showed the preference for the novel object normally. n = 15 for WT, n = 13 for heterozygous mutant. *p<0.05, **p<0.01. Repeated measures ANOVA.
(PDF)

S7 Fig Behavioral analysis of *Nlgn1* P89L homozygote (L/L) mice. (A-C) Three-chamber social interaction test. (A) Time spent in each chamber. A stranger mouse was placed in one of the side chambers in a wired cage, and an empty wired cage was placed in the opposite chamber. (B) Time spent around the inanimate cage and the cage with a stranger. (C) Social preference index of time interaction was identical between WT and *Nlgn1* P89L (L/L) mice. *p<0.05, **p<0.01. Two-way repeated measures ANOVA (A, B), t-test (C), n = 15 for WT, n = 13 for homozygous mutant. (D) Caged social interaction test in the open field. The test consists of two sessions, a 10-min habituation, followed by a 10-min test. Time spent around the cage during the habituation phase with an empty cage and time spent around the cage with an age-matched unfamiliar male mouse in a test session was identical between genotypes. t-test, n = 19 for WT, n = 21 for homozygous mutant. (E) Wins frequency in the social dominance tube test. *Nlgn1* P89L (L/L) homozygote mice had a significantly lower winning rate. *p<0.05. Chi-square test. n = 18 for WT, n = 18 for homozygous mutant. (F) The number of ultrasonic vocalizations emissions at postnatal day 7 induced by maternal-separation during a 5-min session. n = 13 for WT, n = 21 for homozygous mutant. (G, H) Morris water maze to assess hippocampal-dependent spatial learning and memory. On day 1 and 2, mice were trained to find a visible platform in the water maze. On day 3 to 11, mice were trained to find a hidden platform. On day 12, spatial memory was assessed with the platform removed as a probe test (G) Quantification of latency to reach goal across days of training session from day 1 to 11. *p<0.05 Two-way repeated measures ANOVA. (H) Quantification of time spent in each quadrant in a probe test session at day 11. TAR, ADJ and OPP indicates the target, adjacent and opposite quadrant, respectively. *p<0.05, **p<0.01, one-way ANOVA followed by Tukey-Kramer’s multiple comparisons test. n = 19 for WT, n = 21 for homozygous mutant. Data are represented as means ± S.E.M.
(PDF)

S1 Table Seven candidate variants shared by two siblings with ASD (AU072904, AU072905). Candidates with *in silico* evaluations are shown. D, damaging; PD, possibly damaging; T, tolerated; B, benign; H, high; M, middle; L, low; N, neutral.
(XLSX)

S2 Table The list of analyzed off-target candidates selected from CRISPR design tool (http://crispr.mit.edu/). Six candidate sequences with highest-score in exonic region, and nine candidate sequences with highest-score in intronic or intergenic region were analyzed. No off-target was detected.
(XLSX)

S3 Table Statistical analysis of behavior tests in *Nlgn1* P89L heterozygous mice. (XLSX)

S4 Table Statistical analysis of behavior tests in *Nlgn1* P89L homozygous mice. (XLSX)
