# Correction: Functional significance of rare neuroligin 1 variants found in autism

**DOI:** 10.1371/journal.pgen.1007035

**Published:** 2017-10-03

**Authors:** Moe Nakanishi, Jun Nomura, Xiao Ji, Kota Tamada, Takashi Arai, Eiki Takahashi, Maja Bućan, Toru Takumi

In Fig 5, panel A, the labeling of “WT allele” is displaced. In Fig 6, panel H, the labeling “WT(P/L)” is incorrect and should read “WT(P/P)”. In S7 Fig, panel D, the P value of 705 is incorrect and should read “P = 0.705”. There is also an error in the caption for S7 Fig, panel H. The first sentence “Quantification of time spent in each quadrant in a probe test session at day 11” should read “Quantification of time spent in each quadrant in a probe test session at day 12.” Please view the correct Fig 5, Fig 6, S7 Fig and S7 Fig caption below.

**Fig 5:**
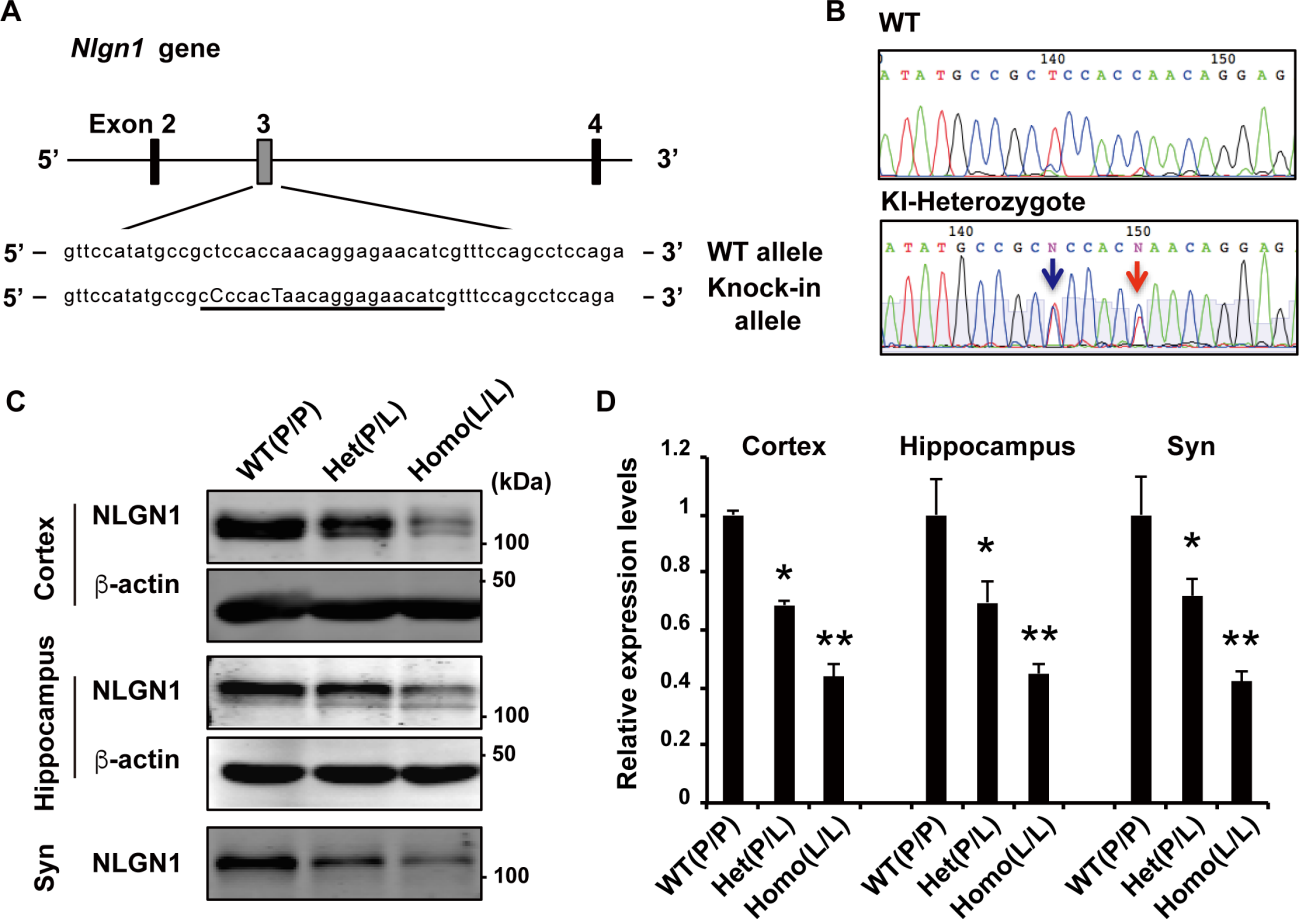
Generation of *Nlgn1* P89L KI mice. (A) Schematic of the mouse genomic locus of *Nlgn1* showing the target site of Cas9. sgRNA sequence is underlined, and replaced bases in knock-in mice are capitalized. (B) DNA sequence electropherograms of WT and knock-in heterozygote mouse. Red arrow indicates the amino acid substitution from proline to leucine (CCA to CTA of residue 89), and blue arrow indicates a silent mutation for genotyping using restriction enzyme *Bsr*BI. (C-D) Western blots of cortex, hippocampus, and cortical synaptosomal fractions from wild-type, *Nlgn1* P89L heterozygote, and homozygote mutant mice. (WT n = 3, heterozygote n = 4, homozygote n = 3) Data are represented as means ± S.E.M. (*p<0.05, **p<0.01 Tukey-Kramer’s multiple comparisons test).

**Fig 6:**
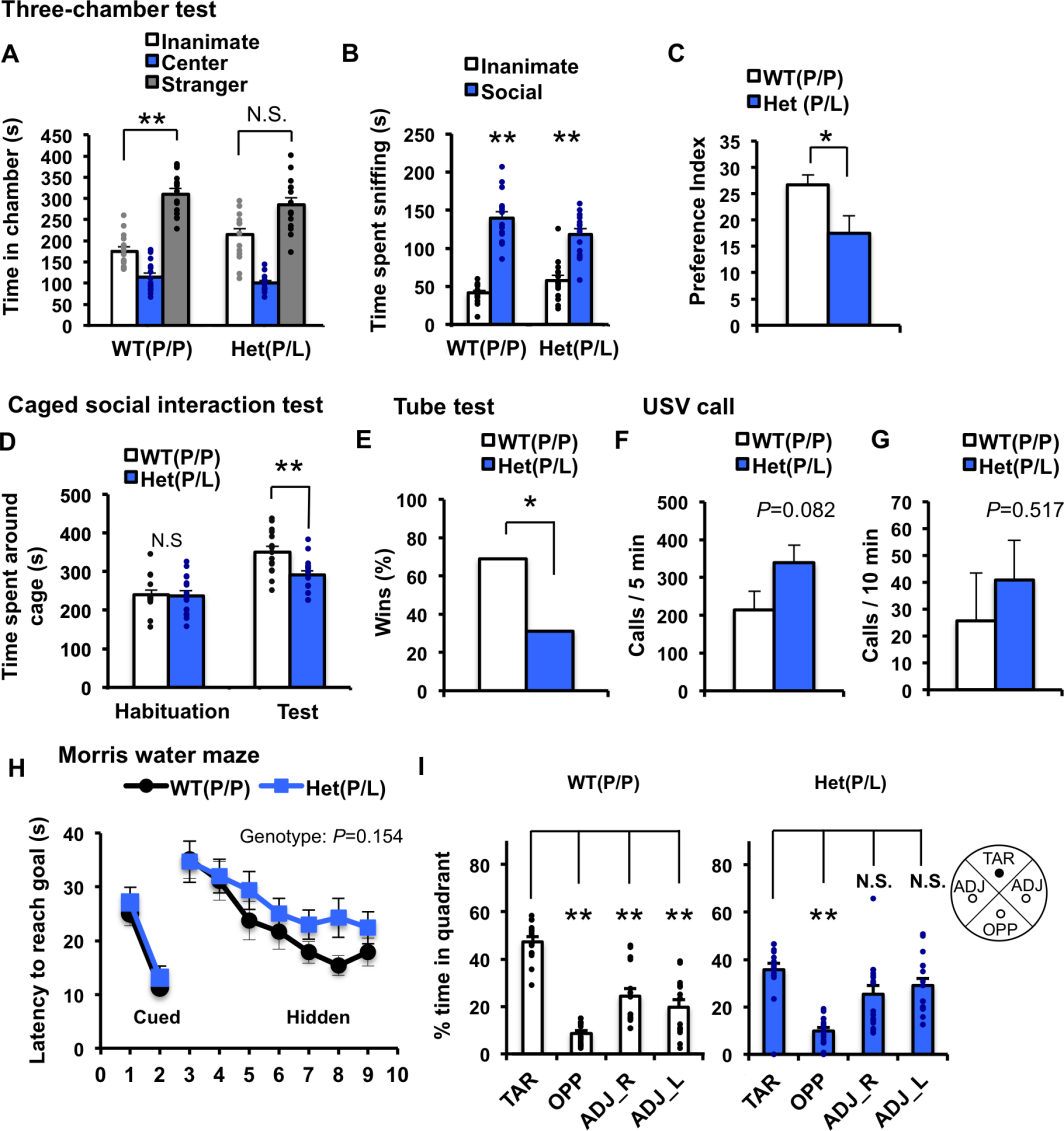
Comprehensive behavioral analysis of *Nlgn1* P89L mice. (A-C) Three-chamber social interaction test. (A) A stranger mouse was placed in one of the side chambers in a wired cage, and an empty wired cage was placed in the opposite chamber. Time spent in each chamber. (B) Time spent sniffing the stranger or the inanimate object. Both WT and *Nlgn1* P89L (P/L) mice spent more time sniffing the stranger than the empty cage. (C) Social preference index was lower in *Nlgn1* P89L (P/L) mice. Preference index = ((Time sniffing the stranger/ (Time sniffing the stranger + Time sniffing the inanimate)) x 100) - 50. *p<0.05, **p<0.01. Two-way repeated measures ANOVA, Bonferroni post-test (A, B). t-test (C), n = 15 for WT, n = 15 for heterozygous mutant. (D) Caged social interaction test in the open field. The test consists of two sessions, a 10 min habituation, followed by a 10-min test. Time spent around the cage during the habituation phase with an inanimate cage was identical between genotypes, however, time spent around the cage with an age-matched unfamiliar male mouse in the test session was significantly less in *Nlgn1* P89L hetrozygote (P/L) mice compared to WT. **p<0.01. t-test, n = 15 for WT, n = 16 for heterozygous mutant. (E) Wins frequency in the social dominance tube test. *Nlgn1* P89L (P/L) heterozygote mice had a lower winning rate. *p<0.05. Chi-square test. (F) The number of ultrasonic vocalizations emissions at postnatal day 7 induced by maternal-separation during 5 min session. (G) The number of ultrasonic vocalizations emissions from adult male mice during 10 min session. Adult female mouse was presented as stimuli. (H, I) Morris water maze to assess hippocampal-dependent spatial learning and memory. On day 1 and 2, mice were trained to find a visible platform in the water maze. On day 3 to 9, mice were trained to find a hidden platform. On day 10, spatial memory was assessed with the platform removed as a probe test (H) Quantification of latency to reach goal across days of training session from day 1 to 9. Two-way repeated measures ANOVA. (I) Quantification of time spent in each quadrant in a probe test session at day 10. TAR, ADJ and OPP indicates the target, adjacent, and opposite quadrant, respectively. WT showed significant preference for the target quadrant than the other quadrants, whereas *Nlgn1* P89L (P/L) mice do not show the preference for the target quadrant compared to adjacent quadrants. **p<0.001, t-test. n = 15 for WT, n = 16 for heterozygous mutant. Data are represented as means ± S.E.M. See S3 Table for all statistics.

## Supporting information

S7 Fig Behavioral analysis of *Nlgn1* P89L homozygote (L/L) mice. (A-C) Three-chamber social interaction test. (A) Time spent in each chamber. A stranger mouse was placed in one of the side chambers in a wired cage, and an empty wired cage was placed in the opposite chamber. (B) Time spent around the inanimate cage and the cage with a stranger. (C) Social preference index of time interaction was identical between WT and *Nlgn1* P89L (L/L) mice. *p<0.05, **p<0.01. Two-way repeated measures ANOVA (A, B), t-test (C), n = 15 for WT, n = 13 for homozygous mutant. (D) Caged social interaction test in the open field. The test consists of two sessions, a 10-min habituation, followed by a 10-min test. Time spent around the cage during the habituation phase with an empty cage and time spent around the cage with an age-matched unfamiliar male mouse in a test session was identical between genotypes. t-test, n = 19 for WT, n = 21 for homozygous mutant. (E) Wins frequency in the social dominance tube test. *Nlgn1* P89L (L/L) homozygote mice had a significantly lower winning rate. *p<0.05. Chi-square test. n = 18 for WT, n = 18 for homozygous mutant. (F) The number of ultrasonic vocalizations emissions at postnatal day 7 induced by maternal-separation during a 5-min session. n = 13 for WT, n = 21 for homozygous mutant. (G, H) Morris water maze to assess hippocampal-dependent spatial learning and memory. On day 1 and 2, mice were trained to find a visible platform in the water maze. On day 3 to 11, mice were trained to find a hidden platform. On day 12, spatial memory was assessed with the platform removed as a probe test (G) Quantification of latency to reach goal across days of training session from day 1 to 11. *p<0.05 Two-way repeated measures ANOVA. (H) Quantification of time spent in each quadrant in a probe test session at day 12. TAR, ADJ and OPP indicates the target, adjacent and opposite quadrant, respectively. *p<0.05, **p<0.01, one-way ANOVA followed by Tukey-Kramer’s multiple comparisons test. n = 19 for WT, n = 21 for homozygous mutant. Data are represented as means ± S.E.M.
(TIF)
