# Supplementary figures and images for: Functional significance of rare neuroligin 1 variants found in autism
Source: PLoS Genet. 2017 Aug 25;13(8):e1006940. doi: 10.1371/journal.pgen.1006940 (PMC5571902; doi:10.1371/journal.pgen.1006940)

**Fig. S1**

**A**

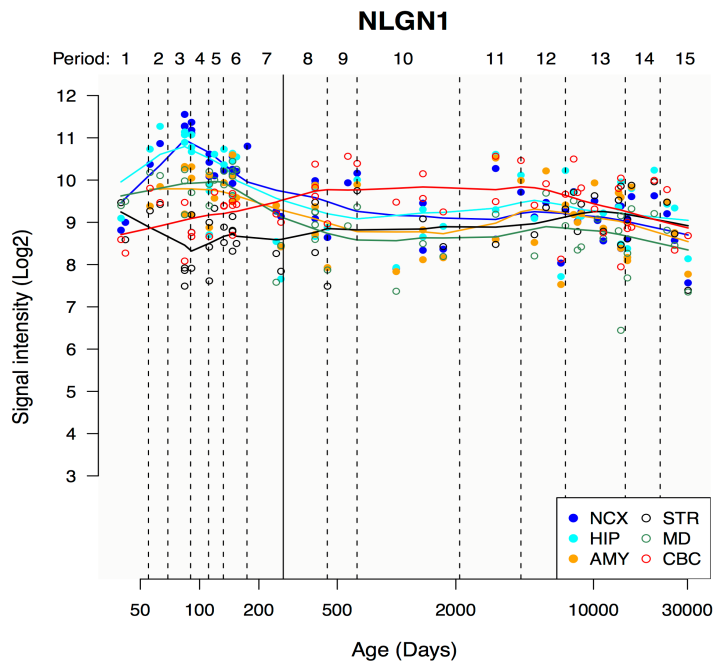

**B**

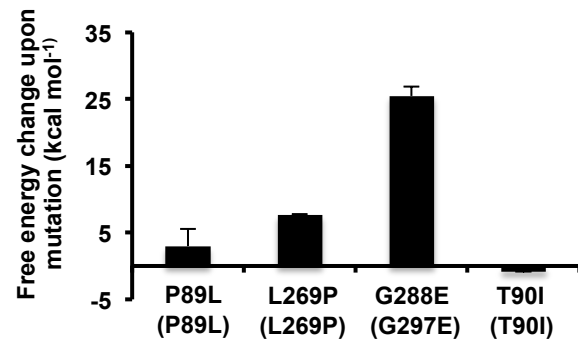

Supplement: S1 Fig — (A) NLGN1 expression in human brain from prenatal to postnatal stages according to Human Brain Transcriptome database (http://hbatlas.org/). Full line indicates birth. NCX: neocortex, HIP: hippocampus, AMY: amygdala, STR: striatum, MD: midbrain, CBC: cerebellar cortex. (B) Calculated result of free energy changes by FoldX. Calculation was performed three times independently. Data represents mean ± S.D. (PDF) [file pgen.1006940.s001.pdf]

Fig. S2

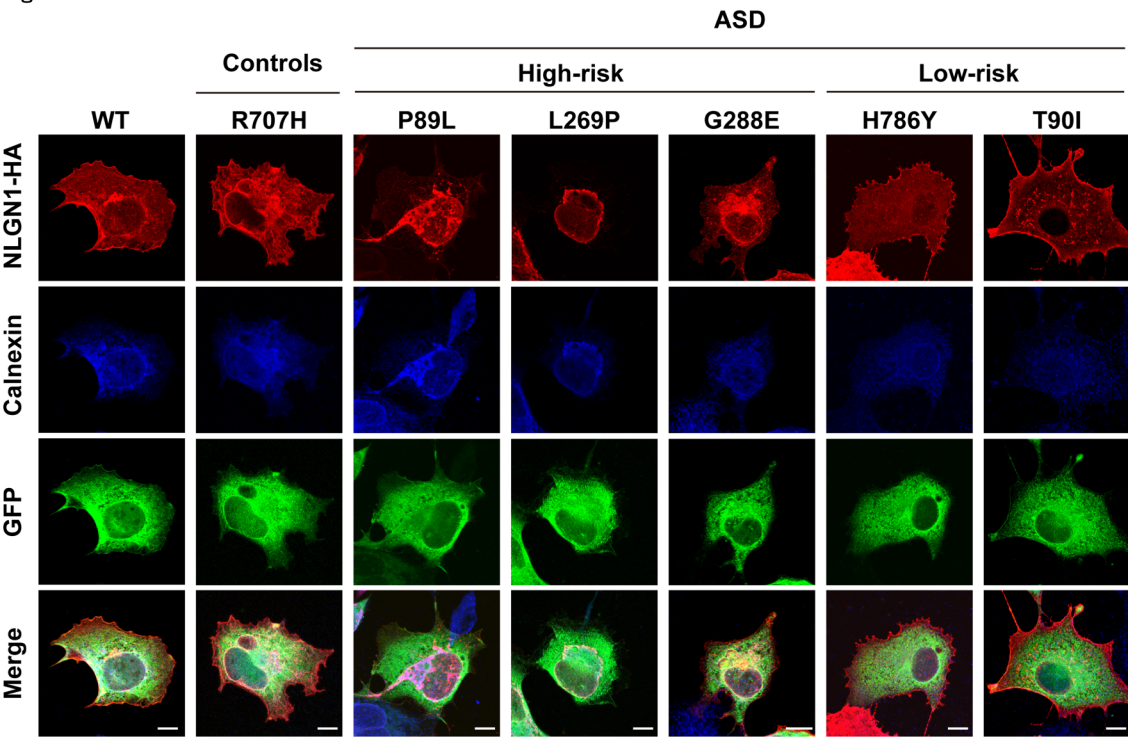

Supplement: S2 Fig — Representative fluorescence images of COS7 cells transfected WT or mutant NLGN1 with HA-tag. GFP was co-transfected to visualize cell shape. Scale bar indicates 10 μm. (PDF) [file pgen.1006940.s002.pdf]

Fig. S3

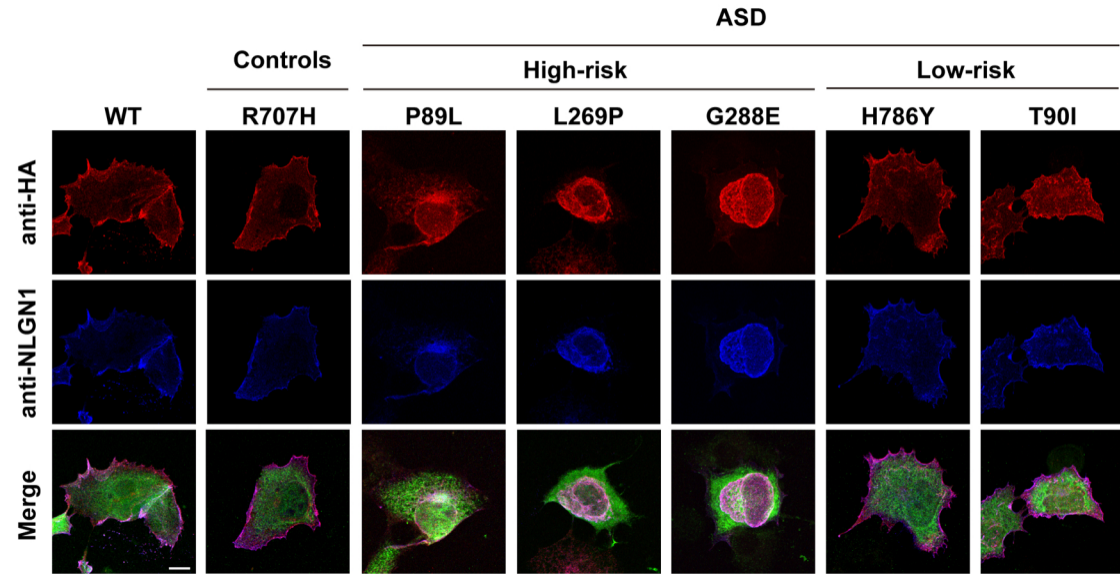

Supplement: S3 Fig — Representative fluorescence images of COS7 cells transfected WT or mutant NLGN1 with HA-tag. NLGN1 sub-localization was detected by anti-HA antibody or anti-NLGN1 antibody, respectively. The signals from two antibodies were almost perfectly merged. GFP was co-transfected to visualize the cell shape. Scale bar indicates 10 μm. (PDF) [file pgen.1006940.s003.pdf]

**Fig. S4**

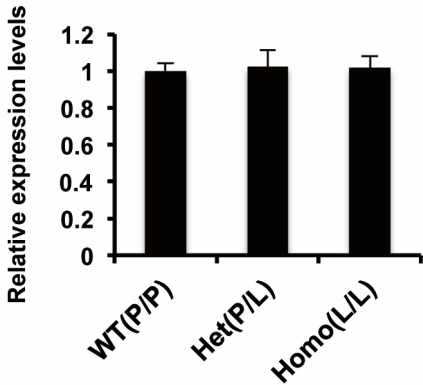

Supplement: S4 Fig — Relative Nlgn1 mRNA expression in the cortex. n = 3 for WT (P/P), n = 5 for heterozygous mutant (P/L), n = 3 for homozygous mutant (L/L). Data represents mean ± S.E.M. (PDF) [file pgen.1006940.s004.pdf]

Fig. S5

**A**

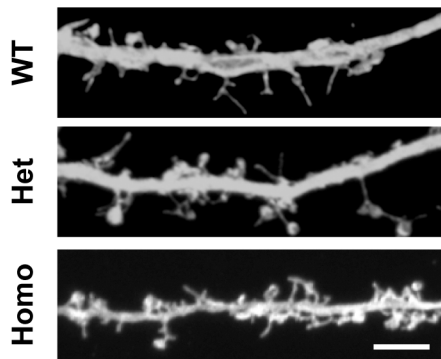

**B**

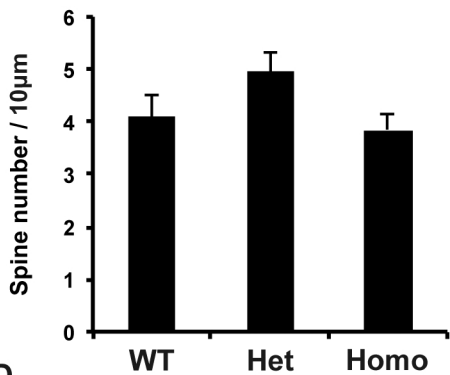

**C**

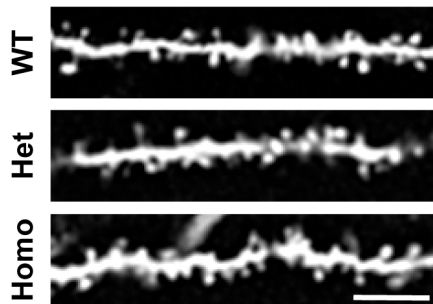

**D**

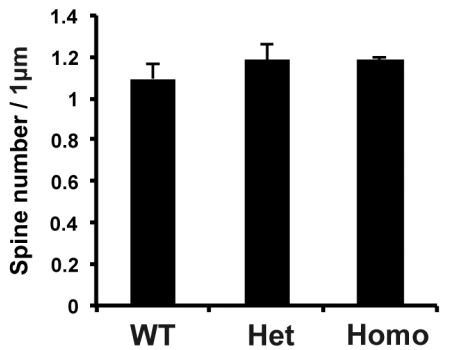

Supplement: S5 Fig — (A) Representative fluorescence images of primary hippocampal neurons (DIV14) collected by WT, Nlgn1 P89L heterozygotes, and homozygote embryos. GFP was transfected to visualize the cell shape. (B) Quantification of the number of spines. No difference was observed among genotypes. n = 3 for WT, n = 5 for Nlgn1 heterozygotes, n = 3 for Nlgn1 homozygotes. (C) Representative images of apical dendrites in hippocampus CA1 from WT, Nlgn1 P89L heterozygotes, and homozygote mice. WT or Nlgn1 P89L mutant mice was crossed with Thy1-YFP line H transgenic strain, and hippocampal neurons were sparsely labeled by YFP. n = 3 for each genotype. (D) Quantification of the number of spines. No difference was observed. Data represent mean ± S.E.M. Scale bar indicates 5 μm. (PDF) [file pgen.1006940.s005.pdf]

**Fig.S6**

**A**

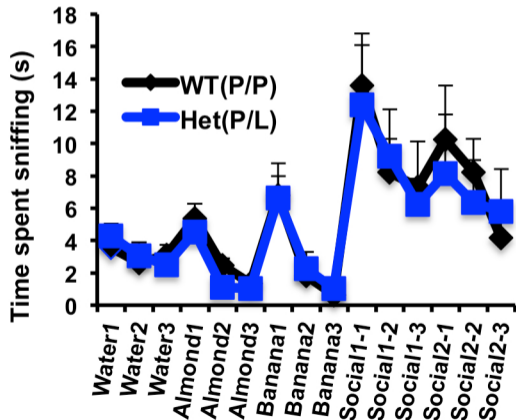

**B**

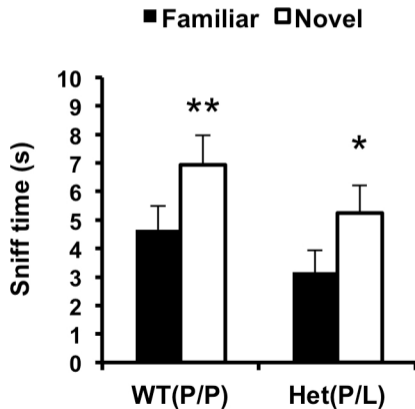

Supplement: S6 Fig — (A) Olfactory habituation/dishabituation test. The olfactory habituation and dishabituation responses to sequential presentations of water, two nonsocial odors (Almond / Banana), and two social odors. Nlgn1 P89L heterozygote (P/L) mice showed normal olfactory habituation and dishabituation responses. (B) Novel object recognition test. Both WT and Nlgn1 P89L (P/L) mice showed the preference for the novel object normally. n = 15 for WT, n = 13 for heterozygous mutant. *p<0.05, **p<0.01. Repeated measures ANOVA. (PDF) [file pgen.1006940.s006.pdf]

### Three-chamber test

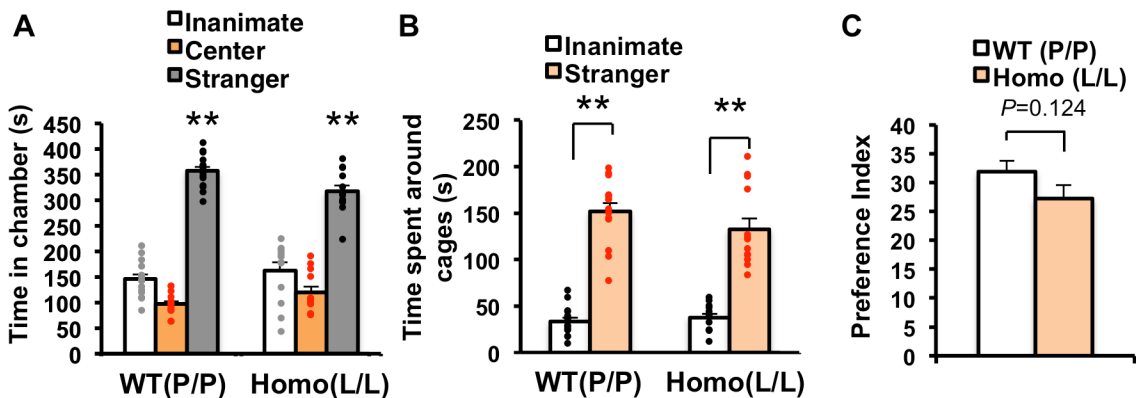

### Caged social interaction test

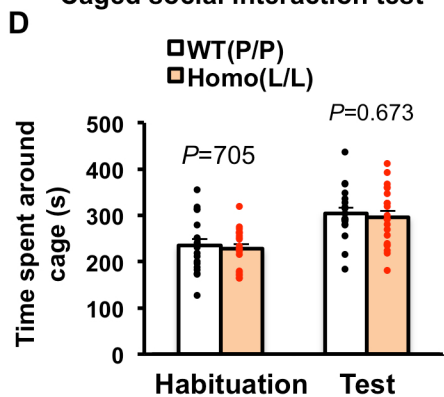

### Tube test

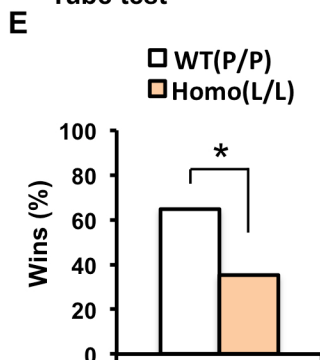

### USV call

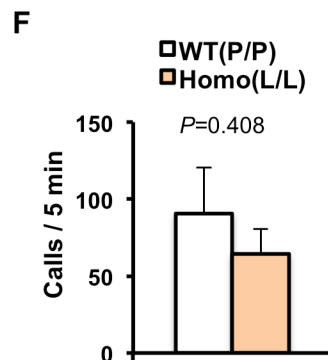

### Morris water maze

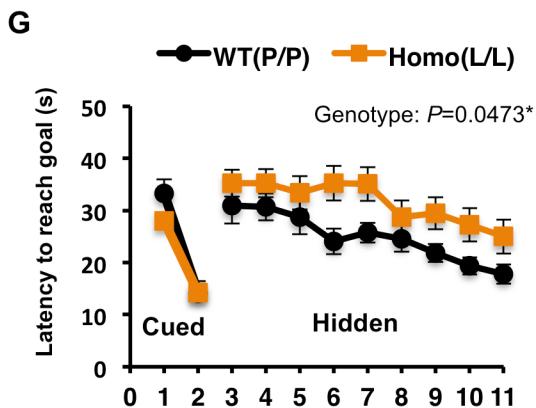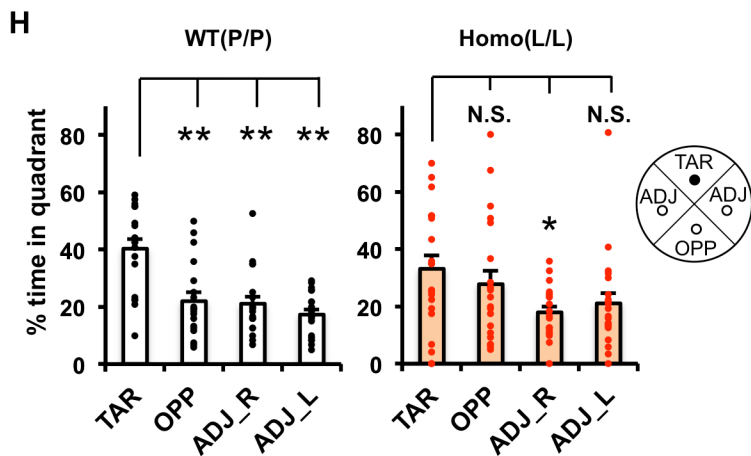

Supplement: S7 Fig — (A-C) Three-chamber social interaction test. (A) Time spent in each chamber. A stranger mouse was placed in one of the side chambers in a wired cage, and an empty wired cage was placed in the opposite chamber. (B) Time spent around the inanimate cage and the cage with a stranger. (C) Social preference index of time interaction was identical between WT and Nlgn1 P89L (L/L) mice. *p<0.05, **p<0.01. Two-way repeated measures ANOVA (A, B), t-test (C), n = 15 for WT, n = 13 for homozygous mutant. (D) Caged social interaction test in the open field. The test consists of two sessions, a 10-min habituation, followed by a 10-min test. Time spent around the cage during the habituation phase with an empty cage and time spent around the cage with an age-matched unfamiliar male mouse in a test session was identical between genotypes. t-test, n = 19 for WT, n = 21 for homozygous mutant. (E) Wins frequency in the social dominance tube test. Nlgn1 P89L (L/L) homozygote mice had a significantly lower winning rate. *p<0.05. Chi-square test. n = 18 for WT, n = 18 for homozygous mutant. (F) The number of ultrasonic vocalizations emissions at postnatal day 7 induced by maternal-separation during a 5-min session. n = 13 for WT, n = 21 for homozygous mutant. (G, H) Morris water maze to assess hippocampal-dependent spatial learning and memory. On day 1 and 2, mice were trained to find a visible platform in the water maze. On day 3 to 11, mice were trained to find a hidden platform. On day 12, spatial memory was assessed with the platform removed as a probe test (G) Quantification of latency to reach goal across days of training session from day 1 to 11. *p<0.05 Two-way repeated measures ANOVA. (H) Quantification of time spent in each quadrant in a probe test session at day 11. TAR, ADJ and OPP indicates the target, adjacent and opposite quadrant, respectively. *p<0.05, **p<0.01, one-way ANOVA followed by Tukey-Kramer’s multiple comparisons test. n = 19 for WT, n = 21 for homozygous mutant. [file pgen.1006940.s007.pdf]
