# Supplementary figures and images for: Correction: Functional significance of rare neuroligin 1 variants found in autism
Source: PLoS Genet. 2017 Oct 3;13(10):e1007035. doi: 10.1371/journal.pgen.1007035 (PMC5626024; doi:10.1371/journal.pgen.1007035)

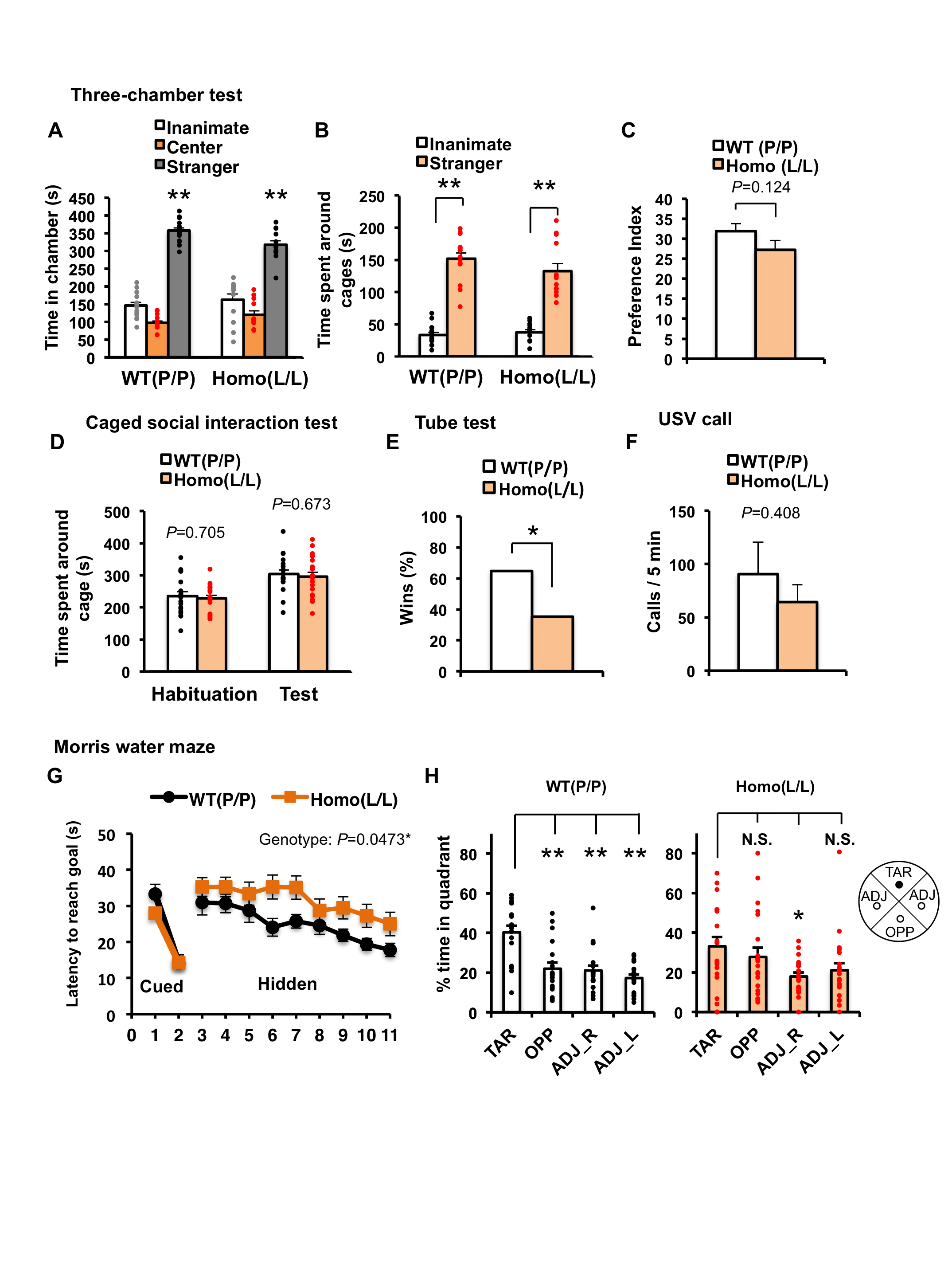

Supplement: S7 Fig — (A-C) Three-chamber social interaction test. (A) Time spent in each chamber. A stranger mouse was placed in one of the side chambers in a wired cage, and an empty wired cage was placed in the opposite chamber. (B) Time spent around the inanimate cage and the cage with a stranger. (C) Social preference index of time interaction was identical between WT and Nlgn1 P89L (L/L) mice. *p<0.05, **p<0.01. Two-way repeated measures ANOVA (A, B), t-test (C), n = 15 for WT, n = 13 for homozygous mutant. (D) Caged social interaction test in the open field. The test consists of two sessions, a 10-min habituation, followed by a 10-min test. Time spent around the cage during the habituation phase with an empty cage and time spent around the cage with an age-matched unfamiliar male mouse in a test session was identical between genotypes. t-test, n = 19 for WT, n = 21 for homozygous mutant. (E) Wins frequency in the social dominance tube test. Nlgn1 P89L (L/L) homozygote mice had a significantly lower winning rate. *p<0.05. Chi-square test. n = 18 for WT, n = 18 for homozygous mutant. (F) The number of ultrasonic vocalizations emissions at postnatal day 7 induced by maternal-separation during a 5-min session. n = 13 for WT, n = 21 for homozygous mutant. (G, H) Morris water maze to assess hippocampal-dependent spatial learning and memory. On day 1 and 2, mice were trained to find a visible platform in the water maze. On day 3 to 11, mice were trained to find a hidden platform. On day 12, spatial memory was assessed with the platform removed as a probe test (G) Quantification of latency to reach goal across days of training session from day 1 to 11. *p<0.05 Two-way repeated measures ANOVA. (H) Quantification of time spent in each quadrant in a probe test session at day 12. TAR, ADJ and OPP indicates the target, adjacent and opposite quadrant, respectively. *p<0.05, **p<0.01, one-way ANOVA followed by Tukey-Kramer’s multiple comparisons test. n = 19 for WT, n = 21 for homozygous mutant. [file pgen.1007035.s001.tif]
